# Heat Shock Alters the Proteomic Profile of Equine Mesenchymal Stem Cells

**DOI:** 10.3390/ijms23137233

**Published:** 2022-06-29

**Authors:** Ahmad Abd-El-Aziz, Angela Riveroll, Blanca Esparza-Gonsalez, Laurie McDuffee, Alejandro M. Cohen, Adam L. Fenech, William J. Montelpare

**Affiliations:** 1 Department of Applied Human Sciences, University of Prince Edward Island, Charlottetown, PE C1A 4P3, Canada; wmontelpare@upei.ca; 2 Atlantic Veterinary College, University of Prince Edward Island, Charlottetown, PE C1A 4P3, Canada; anriveroll@upei.ca (A.R.); besparzagons@upei.ca (B.E.-G.); lmcduffee@upei.ca (L.M.); 3 Biological Mass Spectrometry Core Facility, Faculty of Medicine, Dalhousie University, Halifax, NS B3H 0A8, Canada; alejandro.cohen@dal.ca; 4 School of Climate Change and Adaptation, University of Prince Edward Island, Charlottetown, PE C1A 4P3, Canada; afenech@upei.ca

**Keywords:** heat shock, proteomics, tandem mass tag (TMT) labelling, mesenchymal stem cells, cell signaling, cell differentiation

## Abstract

The aim of this research was to determine the impact of heat stress on cell differentiation in an equine mesenchymal stem cell model (EMSC) through the application of heat stress to primary EMSCs as they progressed through the cell specialization process. A proteomic analysis was performed using mass spectrometry to compare relative protein abundances among the proteomes of three cell types: progenitor EMSCs and differentiated osteoblasts and adipocytes, maintained at 37 °C and 42 °C during the process of cell differentiation. A cell-type and temperature-specific response to heat stress was observed, and many of the specific differentially expressed proteins were involved in cell-signaling pathways such as Notch and Wnt signaling, which are known to regulate cellular development. Furthermore, cytoskeletal proteins profilin, DSTN, SPECC1, and DAAM2 showed increased protein levels in osteoblasts differentiated at 42 °C as compared with 37 °C, and these cells, while they appeared to accumulate calcium, did not organize into a whorl agglomerate as is typically seen at physiological temperatures. This altered proteome composition observed suggests that heat stress could have long-term impacts on cellular development. We propose that this in vitro stem cell culture model of cell differentiation is useful for investigating molecular mechanisms that impact cell development in response to stressors.

## 1. Introduction

Increased periods and severity of extreme heat have become increasingly common because of climate change [1]. In humans, a body temperature above 38 °C can exacerbate the conditions of heat stress, causing an individual to transition from heat discomfort through to heat exhaustion (extreme fatigue), heat syncope (fainting), or to heat stroke, which is a life-threatening condition [2]. Signs and symptoms of heat-related stress can include excessive sweating as well as cognitive impairment [3]. Above 40.6 °C, the heat stress condition is termed “heatstroke”, which leads to an increased risk of loss of consciousness, death, and organ failure [4].

Pregnant women and the unborn fetus are known to be more vulnerable to extreme heat events, due to the impact of pregnancy on thermoregulation [5]. A 2017 meta-analysis by Kuehn and McCormick on the effects of heat exposure on maternal and fetal health found a large variety of negative outcomes in neonates caused by heat exposure, including low birth weights, preterm births, and stillbirths across a wide range of temperature extremes and intervals [6]. The authors concluded that existing heat health recommendations were inadequate, as they failed to prevent heat-related deaths in pregnant women and their fetuses.

Research supports the link between the chronic effects of fetal health and the development of chronic disease in adults, which is a condition of the developmental origins of disease (DoD) hypothesis. The DoD hypothesis, also known as the fetal origins hypothesis, proposed by Barker, states that the effects on the development of the fetus during pregnancy has long-term impacts on an individual’s health [7]. Specifically, the environment of the developing fetus combined with the neonatal environment, to which the newborn is exposed to within the first 2000 days, has a direct impact on the risk factors for developing chronic diseases in adult years.

The effects of heat stress may impact the epigenome, which is defined as the impact of environmental factors on gene expression. Given that such stress can influence epigenetic effects, and these effects can influence gene expression, it is plausible that specific stressors can alter the expression of proteins during the developmental stages of life and, subsequently, influence the future health status. Previous research has shown that environmental stress on the mother during pregnancy can affect the health outcomes of the child [8,9,10]. However, most of these studies were cohort studies in vivo, which limited the conclusions that may have been drawn, due to the complex nature of the stress in the body, the relatively small sample size of the cohort, and a lack of a controlled environment. A cellular model of heat stress on development is warranted to identify these impacts. The differentiation of adult stem cells was identified as a potential model of development, as they are the longest living cells in the adult body that can proliferate and differentiate [11]. Equine MSC cell differentiation was selected as an animal model due to the previous characterization of these cells and their in vitro differentiation by our lab [12].

The objective of this study was to create a mammalian in vitro model to determine the impact of heat as a stress on stem cell differentiation and the resulting protein levels. A proteomic analysis was performed using mass spectrometry to compare relative protein abundances among three cell types, progenitor mesenchymal stem cells (MSCs), differentiated osteoblasts, and adipocytes, cultured at two temperatures, 37 °C and 42 °C, during the process of cell differentiation. The survival of the cells was validated using cell staining and the observation of the cellular differentiation process. The heat stress response was validated with Western blotting using an anti-Hsp90 antibody to quantify an Hsp90 elevation in response to an elevated cell culture temperature. All cell cultures survived at 42 °C, demonstrating higher levels of Hsp90. 

The results indicated that there was a difference in protein levels, determined by a relative abundance across the three cell types when cultured at different temperatures. Many of the differentially expressed proteins were those that are essential to signaling pathways (e.g., Wnt and Notch) known to regulate cell development in vitro and embryonic development in vivo. 

## 2. Results 

### 2.1. Isolation and Differentiation of Stem Cells 

**Figure 1:**
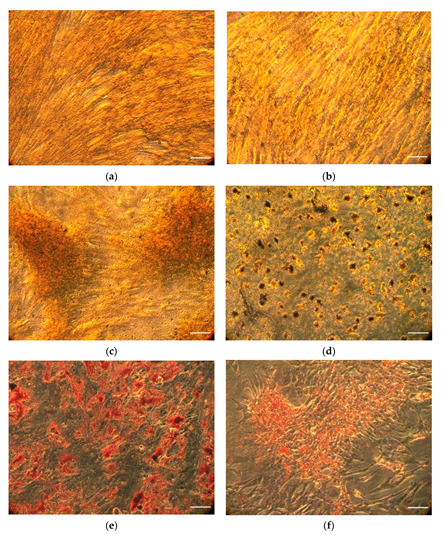
(**a**) MSCs at 37 °C stained with Von Kossa stain. Bar indicates 10 µm. (**b**) MSCs at 42 °C stained with Von Kossa stain. Bar indicates 10 µm. (**c**) Osteoblasts at 37 °C stained with Von Kossa stain. Bar indicates 10 µm. (**d**) Osteoblasts at 42 °C stained with Von Kossa stain. Bar indicates 10 µm. (**e**) Adipocytes at 37 °C stained with Oil Red O stain. Bar indicates 10 µm. (**f**) Adipocytes at 42 °C stained with Oil Red O stain. Bar indicates 10 µm. Cells were grown at 37 °C and 5% CO_2_ in standard media, adipogenic media, and osteogenic media at 37 °C with 5% FBS as described in [12].

MSCs were successfully isolated and expanded from cryopreserved equine adipose tissue. MSCs were able to undergo differentiation to adipogenic and osteoblastic cell lineages indicated by histochemical staining (Figure 1a–f).

The osteoblasts at both temperatures showed calcium deposits when stained with silver nitrate, as well as the formation of tightly knit colonies of cells, compared to the smooth fibroblast-like MSCs that form a uniform monolayer. Oil Red O stained the lipids in adipocyte vacuoles, resulting in red dots. Therefore, the results at 37 °C and 42 °C suggested the cells were capable of differentiating into osteoblasts or adipocytes, despite the stress and despite the heat stress conditions.

### 2.2. Heat Stress Conditions 

**Figure 2:**
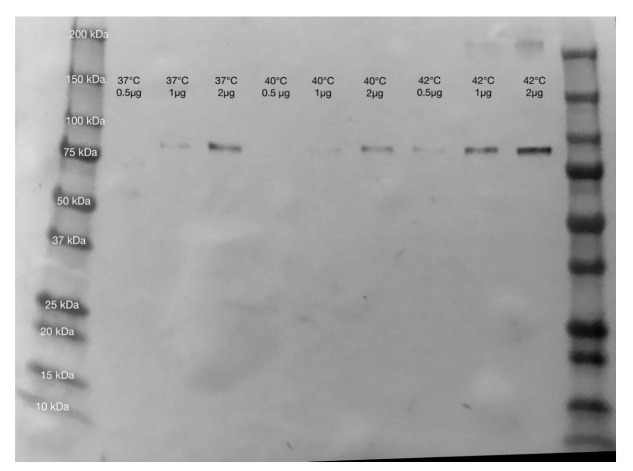
Western blot for HSP90 using anti-HSP90 antibody (HSP90AA1, Aviva Systems Bio (Catalog #: OAED00192)) from osteoblasts grown at 37 °C, 40 °C (not used in final analysis), and 42 °C. Amounts of 0.5, 1, and 2 µg of total protein were used at each temperature to give a gradient for comparison, and all bands showed greater intensity at 42 °C than 37 °C, showing a higher level of HSP90 as a consequence of heat stress.

**Figure 3:**
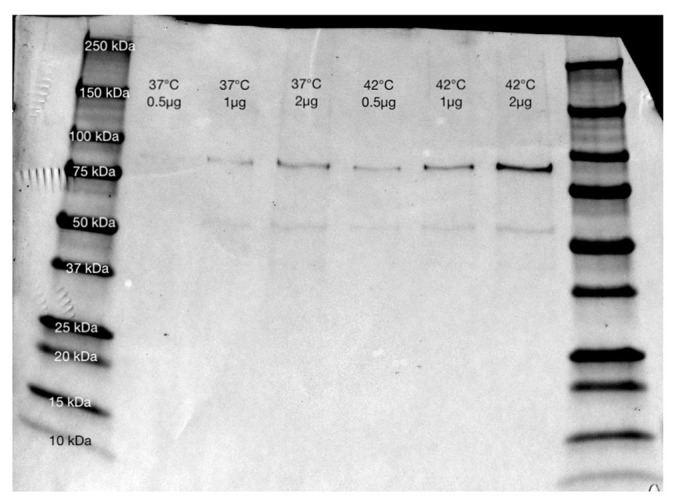
Western blot for HSP90 using anti-HSP90 antibody (HSP90AA1, Aviva Systems Bio (Catalog #: OAED00192)) from adipocytes grown at 37 °C and 42 °C. Amounts of 0.5, 1, and 2µg of total protein were used at each temperature to give a gradient for comparison, and all bands showed greater intensity at 42 °C than 37 °C, showing a higher level of HSP90 as a consequence of heat stress.

Heat stress in cells was confirmed using a Western blot analysis, which showed increased evidence of the heat shock protein HSP90 after 10 days at 42 °C compared with MSCs at 37 °C. Representative Western blots from proteins extracted from adipocytes and osteoblasts at both temperatures are shown in Figure 2 and Figure 3.

### 2.3. Proteomic Analysis using Mass Spectrometry 

The results of the LC-MS/MS provided protein abundance estimates, which showed the differences in protein levels by cell type and temperature conditions. Only 81 proteins showed a cell-specific effect in response to heat stress; that is, while many proteins showed a change in abundance due to either a difference in cell-type or temperature increase, only 81 proteins required both cell-specific differentiation and an elevation in temperature to demonstrate a significant change in protein abundance. For the first twenty proteins that showed this effect, a simple bivariate correlation experiment between the protein abundance in all sample replicates and the pooled samples was then performed for each mass spectrometry experiment. The Pearson correlation coefficients were determined to be either –1.00000 or 1.00000 in all cases, as presented in Tables S1 and S2. This was consistent with what would be expected, as the pools contained all the individual experimental samples, so the variance between samples should have been contained within the variance of the pools.

Heat stress significantly increased the relative abundance of 68 out of 81 proteins expressed at 42 °C in differentiated osteoblasts, leaving a relatively small fraction of proteins (13 out of 81) demonstrating a significantly lower levels at 42 °C compared with 37 °C. In adipocytes, only the protein vimentin (VIM) had a significantly higher levels under heat stress of 42 °C, while 22 out of 81 proteins had significantly lower levels in adipocytes at 42 °C compared with 37 °C. 

#### 2.3.1. Altered Protein Levels of Osteoblasts at 42 °C Compared with Control Cell Population at 37 °C

**Figure 4:**
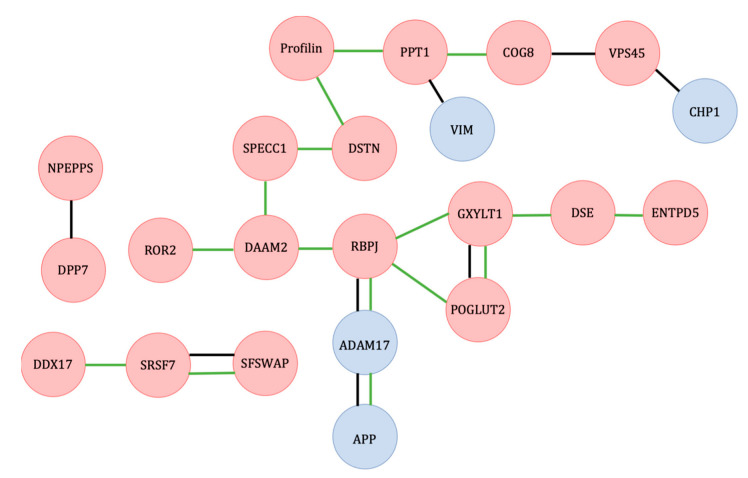
Diagram of functional groupings of proteins with altered levels between 37 °C and 42 °C for osteoblasts. Red—higher level at 42 °C; blue—lower level at 42 °C; black line—protein interactions identified in the STRING database; green—proteins sharing GO tags for biological processes. The functional grouping of the observed proteins was interpreted from documentation in KEGG (Kyoto Encyclopedia of Genes and Genomes) and UniProt databases [13,14]. In addition, the STRING (Search Tool for the Retrieval of Interacting Genes—version 11) database was used to identify protein interactions in existing databases for homologous proteins in humans (*Homo sapiens*) [15]. The labels included in each string diagram refer to the gene names from which the proteins were expressed.

A diagram of proteins with altered levels in osteoblasts in response to heat-stress, organized by related GO tags and STRING database connections, is shown in Figure 4, and a table containing the UniProt accession numbers, protein names, and gene names for the proteins is in Table S3. 

**Figure 5:**
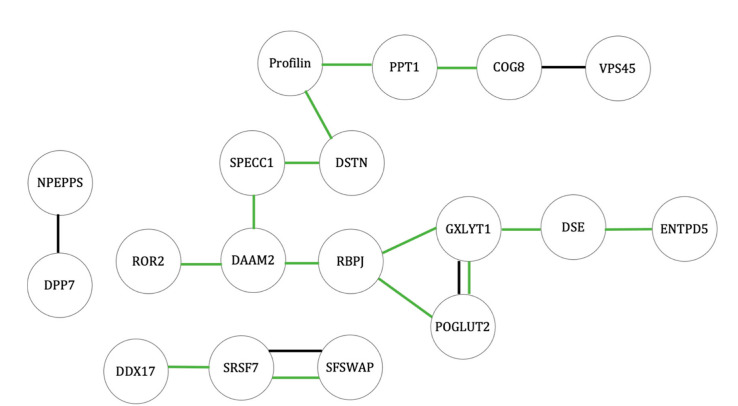
Diagram of functional groupings of proteins with increased protein levels between 37 °C and 42 °C for osteoblasts. Black line—protein interactions identified in the STRING database; green—proteins sharing GO tags for biological processes). Functional grouping of the observed proteins was interpreted from documentation in KEGG (Kyoto Encyclopedia of Genes and Genomes) and UniProt databases [13,14]. In addition, the STRING (Search Tool for the Retrieval of Interacting Genes—version 11) database was used to identify protein interactions in existing databases for homologous proteins in humans (*Homo sapiens*) [15]. The labels included in each string diagram refer to the gene names from which the proteins were expressed.

As the majority (68 out of 81) of these proteins had increased protein levels at 42 °C (Figure 5), it appeared that under heat stress in osteoblasts, an increase in protein levels may be responsible for most of the effects. A table containing the UniProt accession numbers, protein names, and gene names for these proteins is in Table S4.

**Figure 6:**
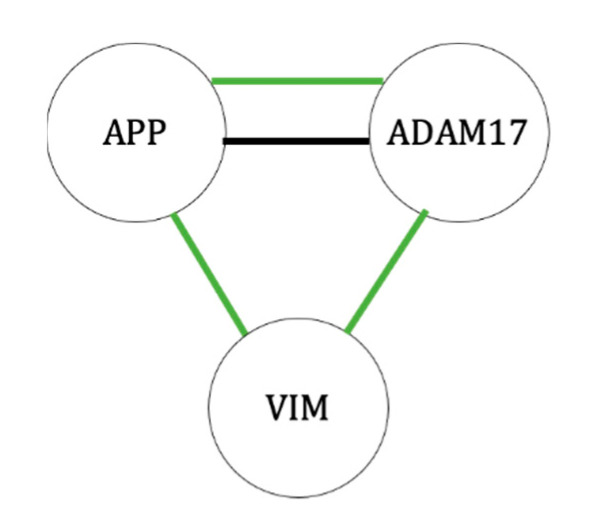
Diagram of functional groupings of proteins with decreased protein levels between 37 °C and 42 °C for osteoblasts. Black line—protein interactions identified in the STRING database; green—proteins sharing GO tags for biological processes. Functional grouping of the observed proteins was interpreted from documentation in KEGG (Kyoto Encyclopedia of Genes and Genomes) and UniProt databases [13,14]. In addition, the STRING (Search Tool for the Retrieval of Interacting Genes—version 11) database was used to identify protein interactions in existing databases for homologous proteins in humans (*Homo sapiens*) [15]. The labels included in each string diagram refer to the gene names from which the proteins were expressed.

Thirteen proteins had lower protein levels at 42 °C compared with 37 °C in osteoblasts and are shown in Figure 6. Table S5 is a table of the UniProt accession numbers, protein names, and gene names for these proteins.

#### 2.3.2. Altered Protein Levels of Adipocytes at 42 °C Compared with Control Cell Population at 37 °C

**Figure 7:**
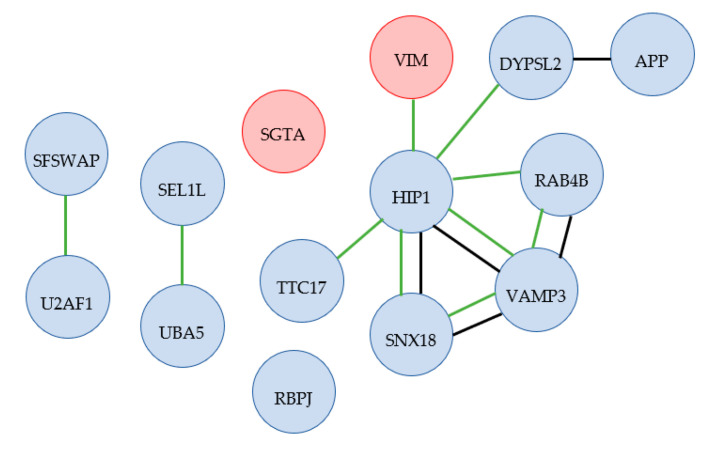
Diagram of functional groupings of proteins with altered protein levels between 37 °C and 42 °C for adipocytes. Red—higher level at 42 °C; blue—lower level at 42 °C; black line—protein interactions identified in the STRING database; green—proteins sharing GO tags for biological processes. Only vimentin (VIM) and SGTA levels were increased under heat. Functional grouping of the observed proteins was interpreted from documentation in KEGG (Kyoto Encyclopedia of Genes and Genomes) and UniProt databases [13,14]. In addition, the STRING (Search Tool for the Retrieval of Interacting Genes—version 11) database was used to identify protein interactions in existing databases for homologous proteins in humans (*Homo sapiens*) [15]. The labels included in each string diagram refer to the gene names from which the proteins were expressed.

A diagram of proteins with altered levels in adipocytes, organized by related GO tags and STRING database connections, is shown in Figure 7, and a table containing the UniProt accession numbers, protein names, and gene names for the proteins is in Table S6. Twenty-two proteins had lower levels in adipocytes at 42 °C compared with 37 °C, and two proteins showed higher levels under the same conditions (SGTA and VIM). 

## 3. Discussion

To construct a model of MSC differentiation under heat stress, MSCs were differentiated into both osteoblasts and adipocytes while being maintained at 37 °C or 42 °C for a duration of 10 days, which was validated with cell staining. All cells were shown to survive under differentiation conditions at 42 °C, and the heat stress was demonstrated with Western blotting, which showed higher levels of HSP90 expression at 42 °C for all cell types.

According to Shimoni et al. [16], heat shock was found to impair MSC proliferation and differentiation in a bovine cell model. Although research by de Magalhães and Passos [17] indicated that MSCs may become senescent, i.e., stop proliferating, and thereby negatively influence the ability to express proteins because of exposure to extreme heat. However, the mechanism of heat shock on MSC differentiation, as opposed to proliferation, is yet unclear.

In our efforts to provide more clarity in explaining the mechanisms of action related to the inhibition of the differentiation and proliferation of MSCs because of exposure to extreme heat, we propose that selected proteins that are part of the Wnt and Notch signaling pathways are plausible candidates to be further investigated in this process.

### 3.1. Wnt and Notch Signaling Balance and Their Effects on MSC Differentiation

In our experiments, the Notch signaling proteins expressed by the genes RBPJ, POGLUT2, GXYLT1, DSE, and ENTPD5 [18] were found to exist at a higher level in osteoblasts that differentiated at 42 °C compared with osteoblasts that differentiated at 37 °C. In osteoblast precursor cells, Notch signaling has been found to suppress osteogenesis via NOTCH2, mediated by the protein RBPJ [19,20]. RBPJ is a DNA-binding protein that regulates the transcription of downstream genes in the Notch pathway specific to the cell type [21]. RBPJ in osteoblasts has not only been shown to inhibit osteogenesis, but this has been shown to prevent osteosclerosis from developing in RBPJ^-^ knockout mice [22]. On the other hand, the overexpression of RBPJ in mouse mesenchymal progenitor cells resulted in increased activity of alkaline phosphatase and a larger number of calcified nodules forming, which suggests that RBPJ is a signal for bone growth as a result of osteoblast differentiation [23].

Although these may seem contradictory, the overall effect of the Notch pathway depends on the stage of the differentiation, namely, that Notch inhibits the initial stages of differentiation in the osteoblasts, but is required for the terminal differentiation in late-stage cells [24]. If this is the case, then its increased expression under heat stress may be driving the continued differentiation towards osteoblasts.

POGLUT2 and GXYLT1 are both proteins involved in O-glycosylation that activates other signaling proteins in the Notch signaling pathway [25,26,27]. POGLUT2, protein O-glucosyltransferase 2, is responsible for the glycosylation of serine 435 in NOTCH1 and NOTCH3 [25]. GXYLT1, glucoside xylosyltransferase 1, adds the first xylose to EGF (epidermal growth factor-like protein), which is then elongated by the protein Fringe, as part of Notch signaling [27]. DSE, dermatan-sulfate epimerase, catalyzes the reaction between chondroitin sulfate and dermatan sulfate, which is a key step upstream of glycosylation processes, and missense mutations in the DSE gene have been found to be a cause of one type of Ehlers-Danlos syndrome [28]. The downstream effect on glycan synthesis has also been demonstrated to cause bone disorders [29]. ENTPD5 (ectonucleoside triphosphate diphosphohydrolase 5 (Inactive)) involved in the folding process of complex proteins through a pattern of glycosylation and deglycosylation that adds and then removes sugar groups for the protein to be folded, helps it adopt a specific conformation [30]. Although not part of the Notch pathway directly, ENTPD5 in Zebrafish is important for skeletal mineralization caused by osteoblasts by controlling the phosphate/pyrophosphate balance [31]. It should be noted that the ENTPD5 that was identified in this study is listed on UniProt (accession no. F7BNQ8) as a possibly inactive equine homolog, so the protein may have an alternative function unknown at this time.

ADAM17 and APP were each expressed at a lower level in osteoblasts at 42°C. ADAM17 and APP were found to be connected in the STRING database as well as both affecting the Notch signaling pathway. ADAM17 (disintegrin and metalloproteinase domain-containing protein 17) breaks down TNF-α and epidermal growth factor receptors (EGFRs) at the surface of the cell [32]. In the Notch signaling pathway, ADAM17 cleaves the cell surface domain of the Notch protein [33]. ADAM17 has been shown to inhibit osteogenesis, unless it is blocked by the protein RUNX2 during differentiation [34]. Therefore, the inhibition of ADAM17 under heat could encourage osteoblast differentiation. APP, amyloid precursor protein, is known to form one of the most common components of amyloid plaques in Alzheimer’s disease, although its biological function is still uncertain. However, it has been discovered to be critical for proper brain development, and this has been hypothesized to be a consequence of neural stem cells [35]. In mice with a knockout of the APP gene, osteoporotic effects were observed as a result of decreased bone formation by osteoblasts [36]. Additionally, APP has been shown to induce the differentiation of glial cells via an interaction with Notch proteins [37]. Although no pattern of interaction was observed in the STRING database, vimentin could also affect the Notch pathway. 

Vimentin (VIM), which is an intermediate filament (type III), a structural protein in the cytoskeleton that has multiple cellular functions, including, but not limited to, cell migration, division, and signaling [38], was also expressed at a lower level in osteoblasts at 42 °C. In mouse vascular smooth muscle cells, shear stress has been shown to result in the phosphorylation of vimentin causing it to bind with the protein Jagged, which results in a mechanical change in Jagged that increases its ability to bind to Notch, therefore, increasing the activation of the Notch signaling pathway [39]. In preosteoblast cells, vimentin inhibits osteoblast differentiation by inhibiting the transcription of osteocalcin [40]. Therefore, decreased levels of vimentin in osteoblasts under heat may be a negative feedback mechanism in the cell to prevent this. Of particular interest, vimentin has also been connected to the process of neuronal differentiation under heat stress conditions [41]. This suggests that, in addition to the MSC differentiation model used in our research, vimentin may play a role in the general response to heat in differentiation processes.

Notch signaling, which can be considered a fundamental developmental process, similarly prevents adipogenic differentiation in preadipocytes, although the pathway is dependent on the NOTCH1 protein, rather than the NOTCH2 isoform [42]. The dysregulation of the Notch pathway has been associated with T-cell acute lymphoblastic leukemia in humans [43].

In the present study, vimentin showed contrasting outcomes under the two stimulus conditions: osteogenesis versus adipogenesis. Whereas we observed a lower level of protein levels in osteoblast differentiation under the heat stress of 42 °C, we observed a higher level of protein levels during differentiation in the formation of adipocytes. This was consistent with work by Lian et al. (2009), which reported that vimentin could inhibit osteoblast differentiation, while Pattabiraman et al. (2020) indicated that vimentin could enhance the differentiation of selected cell types, specifically, neuronal cells in knockout mice. Similarly, the expression of vimentin has been shown to contribute to the formation of lipid droplets in early adipogenesis in humans [44].

In the process of MSC differentiation, the increased expression of vimentin observed in the generation of adipocytes under heat stress would lead to the inhibition of osteogenic differentiation; therefore, we hypothesized that this prioritized the signals that led to adipogenesis.

Wnt signaling pathway-associated proteins identified in this experiment included RBPJ, ROR2, and DAAM2, all of which exhibited increased protein levels at 42 °C in osteoblasts. Further, RBPJ showed decreased protein levels at 42 °C in adipocytes. As stated above, RBPJ is a protein in the Notch signaling pathway; however, it can also form a complex with the Notch protein that can have an inhibitory effect on Wnt signaling, thus, forming a connection between both signaling pathways. ROR2, tyrosine-protein kinase transmembrane receptor ROR2, is involved in bone growth when found on osteoclast precursor cells by controlling Wnt5a bound to osteoblasts signaling with ROR2 [45]. However, as ROR2 is normally found with higher expression in osteoclasts, this could potentially interfere with this signaling process. It is important to note that in our review of the abundant proteins from the exposure to heat stress, the protein disheveled–associated activator of morphogenesis 2 (DAAM2), which controls the formation of complexes with Wnt receptors in the canonical Wnt signaling pathway, could be influenced by exposure to extreme heat. Understanding the role of heat exposure on DAAM2 is important to developmental processes, as this protein is necessary for dorsal patterning, a process during embryo development that controls cell fate based on whether the cells are located on the dorsal or ventral side of the embryo [46].

### 3.2. Cytoskeletal Proteins Necessary for Differentiated Cell Morphology and Cell Signaling

Profilin, DSTN, SPECC1, and HIP1, as well as the previously described DAAM2, are involved in the development of the cytoskeleton, which affects the morphology of the cell as well as cell signal transmission [47]. In the case of MSC differentiation, mechanical forces that induce differentiation signaling are transferred from the cytoskeleton of the cell to the cytoskeleton of the nuclear membrane, which also contains actin through linker complexes [48]. The disruption of the actin cytoskeleton may, therefore, have a profound inhibitory effect on the differentiation of MSCs, which may explain the decrease in the size and uniformity of the cell colonies observed in the microscope image between Figure 1c at 37 °C and Figure 1d at 42 °C. DSTN (destrin) and profilin alter actin polymerization. Destrin, also known as actin depolymerization factor (ADF), is part of the ADF/Cofilin family of proteins, which can sever actin filaments (F-actin). The protein destrin binds to actin monomers (G-actin), interacting with CAP (cyclase-associated protein), which converts ADP (adenosine diphosphate) bound to the G-actin into ATP (adenosine diphosphate) that is then used to polymerize the G-actin into new filaments [49]. Profilin also assists in this nucleotide exchange process, but also binds to a specific groove on the G-actin monomers, allowing them to polymerize into active filaments in a specific direction, instead of to themselves, or to the wrong end of the growing filament [50]. Actin filaments form the structure of the cell, but are also necessary for several types of endocytosis, including clathrin-independent endocytosis, phagocytosis, and macropinocytosis [51].

It is noteworthy that, in the present study, we identified huntingtin-interacting protein 1 (HIP1), which is an actin and clathrin binding protein that is involved in the pathogenesis of Huntington’s disease [52]. Likewise, we also identified a measurable abundance of the protein SPECC1. The mechanism of SPECC1 (also known as Cytospin-b in humans) is not well understood; however, it has been associated with facial cleft formation in zebrafish, suggesting its importance in early development [53].

Several of the proteins found to be less expressed in adipocytes under heat stress are also involved in cytoskeleton organization, which is necessary for the formation of mature adipocytes [54]. The homologous protein to TTC17 API5 in humans, TTC17 (Tetratricopeptide repeat protein 17), is an actin-organizing protein involved in the formation of cilia [55], while DPYSL2, dihydropyrimidinase-related protein 2, is a microtubule-binding protein involved in neuronal differentiation [56].

Observing decreased protein levels of DPYSL2 is important when one considers the concomitant increase in the abundance of vimentin during the differentiation process. Under normal conditions, DPYSL2 controls the expression of vimentin; however, this control process is lost during exposure to heat stress. This can have important implications of disease processes as, according to Rmaileh et al. [57], vimentin has been associated with breast cancer metastasis and thereby may be free to act in this regard during extreme heat.

Huntingtin-interacting protein 1 (HIP1), as mentioned above, is an actin and clathrin binding protein [52]. Clathrin-mediated endocytosis involves clathrin-lined pits that line the plasma membrane, which pinch off to form vesicles, with the clathrin used to bind the molecules of interest (specific to different clathrin chains) to be brought into the cell via endocytosis. This mechanism is required to control signaling proteins on the cell surface, changing the structure plasma membrane due to environmental factors, and regulating the level of transmembrane transporter and receptor proteins on the plasma membrane [58]. HIP1 is also necessary for spermatogenetic progenitor cell differentiation, which suggests the importance of its presence in processes during prenatal development [59].

### 3.3. mRNA Transcription and Splicing Regulation of Differentiation and Stem Cell Proliferation

Proteins SFSWAP, SRSF7, and DDX17 are involved in the formation of mature mRNAs through the control of mRNA transcription and splicing. In particular, SFSWAP and SRSF7 alter the spliceosome by repressing the splicing of exon 10 of the Tau protein [60,61]. Tau has been implicated in the pathogenesis of neurodegenerative diseases, including Parkinson’s disease and Alzheimer’s disease [62]. The alternative splicing of Tau exon 10 causes a dysregulation in the balance of Tau isoforms, which has been shown to be correlated with the development of these neurodegenerative diseases [63]. SRSF7, serine/arginine-rich splicing factor 7, also has been associated with the ubiquitin proteasome pathway through which the Tau protein balance may be affected [61]. However, SFSWAP (splicing factor, suppressor of the white-apricot homolog), which is also known in humans as SRSF8, was identified through homology to other SRSF proteins, so the mechanism is less well understood than SRSF7 [64]. DDX17 (probable ATP-dependent RNA helicase) is a helicase that unwinds DNA and alters the chromatin structure to affect transcriptional regulation [65]. DDX17 is also a key factor for the epithelial–mesenchymal transition, a process that reverses the differentiation of mature epithelial cells back into MSCs. In this transition, DDX17 controls transcription processes, which affect differentiation, as well as a miRNA-negative feedback loop [66].

As with the osteoblasts, the protein levels of RNA splicing proteins (SFSWAP and U2AF1 for the adipocytes) were altered. U2AF1 controls cell fate via the alternative splicing of genes. In general, higher levels of U2AF1 are associated with the ability of stem cells to renew themselves, as well as their pluripotency, while decreased levels have been observed during differentiation [67]. The decreased level in adipocytes under heat stress may, therefore, be as a result of requiring increased signals for the differentiation to take place. 

### 3.4. Endocytosis-Related Proteins in Adipogenic Differentiation 

The group of proteins RAB4B, SNX18, HIP1, and VAMP3 also functions in endocytosis. Endocytosis is a cellular function responsible for the uptake of molecules from outside the cell into vesicles formed from the plasma membrane [68]. RAB4B, Ras-related GTP-binding protein 4b, controls glucose transporter GLUT4 in adipocyte by altering how the glucose transporter protein GLUT4 is either stored in endosome compartments or moved to the plasma membrane, which alters the glucose uptake through adipocyte cells [69].

In adipocytes, vesicles containing GLUT4 are needed for the action of insulin. The interaction of these vesicles with the cell membrane is mediated by SNARE (soluble N-ethylmaleimide-sensitive factor attachment protein receptor) complexes, of which the vesicle SNARE complexes are isoforms of VAMP (vesicle-associated membrane protein). Although VAMP2 has been identified as a major contributor to this interaction, VAMP3 is also involved in SNARE interactions [70]. Additionally, SNX18, sorting nexin 18, which was found to be important in neuron differentiation as its dynamic localization within the cell corresponds to the growth of the axon [71] is also involved in controlling endocytosis at the cell membrane, which is also necessary for cell separation to occur during mitosis [72,73].

### 3.5. Endoplasmic Reticulum-Associated Degradation Affecting Adipocyte Differentiation

UBA5 and SEL1L are involved in ERAD (endoplasmic reticulum-associated degradation), a pathway that connects to the ubiquitin–proteasome system. Ubiquitination (or ubiquitylation) tags proteins with ubiquitin, which can target misfolded proteins for destruction through the ubiquitin–proteasome system (in addition to other functions of ubiquitin) [74]. UBA5 activates UFM1, which activates ERAD, which targets proteins for disposal via ubiquitination [75]. UBA5 is also involved in erythroid (red blood cell) differentiation [76]. Similarly, SEL1L is an adaptor protein involved in ERAD, which has also been shown to be involved in preventing the aggregation of lipoprotein lipase (LPL) [77,78]. As LPL is necessary for the entry of fatty acids into specific tissue cells, including adipocytes, this suggests an important role for the SEL1L protein specific to adipocytes [78,79].

### 3.6. Potential New Protein Targets

It should be noted that, in the proteins with lower levels of protein levels in the heat-stressed osteoblasts, two of the genes coding for these proteins were identified by homology (K9KA63 and F6SUF8), one of which was a fragment, and, therefore, were not well characterized equine proteins. A newer characterization of these proteins could lead to the identification of other mechanisms in this group of proteins. Similarly, SGTA (small glutamine-rich tetratricopeptide repeat containing alpha), which showed higher levels of protein levels in heat-stressed adipocytes, has been found to bind to HSP70 and HSP90, as well as steroid receptors, which regulate certain tissues via steroid hormones [80]. 

### 3.7. Overall Effects 

Of the proteins that had significantly different levels under heat for both osteoblasts and adipocytes, several appeared to be involved in maintaining the differentiation process. Proteins known to be involved in the differentiation of that cell type tended to have increased protein levels under heat. For example, RBPJ was associated with osteoblast differentiation and was more highly expressed in the heat-stressed osteoblasts compared with those grown at 37 °C. On the other hand, those known to cause the inhibition of differentiation tended to show decreased protein levels under heat, thus, preventing the inhibitory signal. Vimentin and ADAM17 have been shown to disfavor osteogenic differentiation in osteoblast precursor cells and were, therefore, less expressed in the heat-stressed osteoblasts. A similar inhibitory effect was observed for adipocytes under heat stress with RAB4B, which inhibited adipogenic differentiation. Although SEL1L knockdown experiments were not found to impact adipogenic differentiation, the SEL1L protein is important for adipocyte function [78]. The loss of SEL1L in mouse embryos was shown to be lethal, suggesting it is necessary in some developmental capacity in the organism [81]. It should be noted that many of these proteins decreased under heat stress were still present in low quantities, implying that the genes were not turned off completely. This is important, as it may explain the survival of the heat-stressed cells, even if they are less capable of lipid uptake compared to unstressed cells.

In addition to proteins involved in MSC differentiation, DPYSL2 and SNX18 are involved in neural differentiation, HIP1 and U2AF1 may be important for the control of differentiation of various progenitor stem cells, and UBA5 has been shown to be necessary for megakaryocyte and erythroid progenitor differentiation, which are necessary for the formation of platelets and erythrocytes (red blood cells), respectively. Although these genes may not have been previously demonstrated to control osteogenic or adipogenic differentiation, the fact that they differed due to heat could be a sign that they are involved in MSC differentiation. Furthermore, it may be that these proteins react similarly in other precursor or stem cells under the same type of stress to maintain those differentiation processes, although this would need to be verified experimentally using specific cell types.

### 3.8. Impact of Heat Stress on Cell Development

While the focus of this cell model is on differentiation, the developmental impacts of these proteins should also be considered. Effects on embryonic development may drastically affect the organism later in life, potentially including some of the results of prenatal stress effects noted by King et al., such as metabolic changes and cognitive development [82,83]. Effects based on heat stress may be tissue-specific. In particular, the effects of heat stress on neuronal development may not be limited to neuron differentiation, but on the developing brain of the newborn. Other developmental impacts include the effects of heat stress on tissue formation. For example, DSE affects glycan synthesis, which is necessary for several functions in mammalian tissue cells, and mutations resulting in the loss of the DSE enzyme function can result in the form of Ehlers-Danlos syndrome, a disease that weakens connective tissues [28].

ROR2 affects bone growth through interactions with an isoform of Wnt in osteoclasts [45]. Although osteoclasts are derived from a different cell lineage than osteoblasts, this shows a potential role for ROR2 in bone tissue development. DAAM2 regulates dorsal patterning, which is important for the development of many organs and tissues, one of which was experimentally identified as the spinal cord. This developmental outcome is critical for the proper growth and function of the nervous system [46]. Finally, SPECC1, which we also observed as demonstrating increased protein levels in the osteoblasts under heat stress, may be necessary for the development of the facial cleft, although the exact mechanism of this gene is unknown [53].

## 4. Materials and Methods

### 4.1. Isolation and Differentiation of Mesenchymal Stem Cells

Equine postnatal mesenchymal stem cells were used in the creation of the in vitro model. Mesenchymal stem cells (MSCs) were previously isolated using enzyme digestion from equine adipose tissue and cryopreserved. Cryopreserved MSCs were thawed and expanded for use in this study. The cells were cultured in standard media with 10% FBS at 37 °C and 5% CO_2_ to third passage, at which time they were used for heat shock assays.

### 4.2. Heat Stress Conditions

Heat shock assays included MSCs at third passage in standard media, adipogenic media, and osteogenic media at 37 °C with 5% FBS as described in [12] as controls, and the same conditions at 42 °C to induce heat stress. All cells were maintained in the specific culture conditions for 10 days. The cells were detached from plastic flasks with 10 mL of a 1:4 dilution of 0.25% Trypsin-EDTA (Gibco Catalog #25200-072, Waltham, MA, USA) to CellStripper Dissociation Reagent (Corning™ 25056CI, Corning, NY, USA) and incubated for 30 min at 37 °C. The cells were subcultured into the second passage. Once confluent, 2.5 × 10^6^ of the mesenchymal stem cells were separated into three conditions based on culture media and grown for 10 days before being harvested. The first condition was standard media with 5% FBS. The second condition was osteogenic media (standard media with β-glycero-phosphate, ascorbic acid, and dexamethasone) to promote differentiation of osteoblasts [12]. The third condition was adipogenic media (standard media with rabbit serum, L-glutamine, insulin, pantothenic acid, biotin, dexamethasone, 3-Isobutylmethylxanthine (IMBX), and rosiglitazone) to promote the differentiation of adipocytes [84].

The cells were harvested by washing each flask 3× with 10 mL PBS, adding 5 mL PBS and scraping, then adding to 1.5 mL Eppendorf tubes, and centrifuging the tubes at 2500× *g* for 10 min, removing the supernatant, and were then stored immediately at −20 °C.

### 4.3. Western Blot

To confirm that cells were stressed because of exposure to 42 °C, a Western blot electrophoretic procedure was used.

Protein was extracted from the cells by addition of 200 µL of Lysis Buffer (10mM Tris HCl at pH 8.0, 100mM NaCl, 1mM EDTA, 2% SDS, 1mM DTT, and 7µL of protease inhibitor (Sigma-Aldrich, Saint Louis, MO, USA, catalog ID: P8340) for 7 × 10^6^ cells). A Fisher Sonic Dismembrator Model 300 was used to lyse the cells and the lysate was then clarified by centrifugation at 5000× *g* for 20 min at 4 °C.

Total protein concentration was determined using BCA to establish the gradient of protein for the Western blot. Thermo Scientific Pierce BCA Protein Assay Kit (Catalog #23225) was used, and absorbance was measured using a SpectraMax^®^ Plus384 plate reader set to 562nm. To reduce the disulfide bonds of extracted protein in lysis buffer, an equivalent amount of 2× Laemmli working solution was added (2× Laemmli Sample Buffer (Bio-Rad, Berkeley, CA, USA, Catalog #: 1610737), 5% β-mercaptoethanol), and the solution was boiled for 5 min.

The protein samples were then loaded onto precast 4–20% polyacrylamide gel (Bio-Rad Mini-PROTEAN^®^ TGX™; catalog #: 4561096) and run using Mini-PROTEAN^®^ Tetra Vertical Electrophoresis Cell for Mini Precast Gels (Bio-Rad, catalog #: 1658005). The gel was then imaged using a Bio-Rad ChemiDoc™ imaging system (Catalog #: 12003153). The gel was blotted onto a nitrocellulose membrane using a Mini Trans-Blot^®^ Electrophoretic Transfer Cell (Catalog #: 1703930) and then stained first with primary antibody (anti-HSP90: HSP90AA1, Aviva Systems Bio (Catalog #: OAED00192), 1:400 dilution) for one hour of incubation, followed by secondary antibodies conjugated to horseradish peroxidase, HRP (Aviva Rabbit Anti-Mouse IgG Antibody: HRP-conjugated (Catalog #: OATA01311), 1:400 dilution)). The nitrocellulose membrane was then washed with 1× TTBS (100 mL 10× TBS, 2.5 mL 10% Tween20 (Bio-Rad, Cat #: 1610781), 500mL ddH_2_O), 1× TBS, and ddH_2_O before being developed by addition of Thermo Scientific™ 1-Step™ Ultra TMB-Blotting Solution (Catalog #: 37574) and imaged using a Bio-Rad ChemiDoc™ imaging system.

Adipogenic differentiation was validated with Oil Red O staining [84] and osteogenic differentiation with Von Kossa staining [85]. All stained cells were observed, and pictures were taken with a Zeiss Axiovert 40 CFL Trinocular Inverted Fluorescence Phase Contrast Microscope.

### 4.4. Proteomic Analysis Using Liquid Chromatography/Tandem Mass Spectrometry (LC-MS/MS)

#### 4.4.1. A Chloroform/Methanol Precipitation Protocol

Proteins were extracted for use in liquid chromatography/tandem mass spectrometry (LC-MS/MS) analysis into lysis buffer as for the Western blot procedure above. Proteins were precipitated using a chloroform/methanol precipitation protocol [86] and protein was stored at −20 °C. To a 200 µL sample of protein in lysis buffer, 800 µL of methanol, 200 µL of chloroform, and 600µL of deionized water were added, mixing thoroughly after each step. This was then centrifuged for 1 min at 14,000× *g*. The top aqueous layer was then removed, and 800 µL of methanol was added and mixed thoroughly. The samples were then centrifuged for 2 min at 14,000× *g*. The methanol layer was removed, and the pellet was allowed to dry. The resulting pellet was resuspended in 8M urea buffer (8M urea, 0.4M NH_4_HCO_3_, 0.1% SDS) and stored at −20 °C.

#### 4.4.2. Reduction and Digestion

The sample concentrations were normalized by diluting all protein samples in urea buffer to have a final volume of 200 µL and 12µg of total protein.

Before trypsin digestion, protein samples were reduced with 10 µL 0.5M DTT (Fisher BioReagents: BP172-5, Pittsburgh, PA, USA) for 30 min at 60 °C. 20 µL of 0.7M iodoacetamide (Sigma: I1149, St. Louis, MO, USA) was then added, and the samples were incubated for 30 min at room temperature. The samples were then diluted with 1.2 mL of Milli-Q^®^ water, then digested with 100 µL of a 0.02 µg/µL buffered trypsin solution (20µg Pierce™ Trypsin Protease (Thermo Scientific: 90657, Waltham, MA, USA), dissolved in 50uL trypsin resuspension dilution buffer (Promega, V542A, Madison, WI, USA) and diluted to 1 mL with water), and incubated at 37 °C overnight. An equivalent amount of trypsin was added the next morning and incubated for an additional two hours at 37 °C.

The digested samples were desalted using the following solid-phase extraction protocol. 1.5 µL of trifluoroacetic acid (TFA) was added to the samples and the pH of all samples was set to 3.0 using formic acid. An Oasis HLB 3cc Extraction Column (Waters™ WAT094226, Milford, MA, USA) was conditioned once with 0.5mL methanol, once with 50% acetonitrile +0.1% TFA, and twice with 1 mL 0.1% TFA. The samples were loaded onto the columns and washed five times with 1 mL of 0.1% TFA. The samples were then eluted off the column twice with 0.5mL of 50% acetonitrile +0.1% TFA and once with 0.5 mL of 80% acetonitrile +0.1% TFA and dried.

#### 4.4.3. Tandem Mass Tag (TMT) Labeling

All protein samples were resuspended in 70 µL 100 mM HEPES buffer (Gibco: 11344041, Wltham, MA, USA). 14 µL of each sample from both sets (proteins from cells grown at 37 °C and proteins from cells grown at 42 °C) was combined and split into two pools with volume of 126 µL to facilitate comparison between both experiments. The TMT^10^plex Isobaric Reagent Set (Thermo Scientific: 90110) and the TMT^11^-131C Label Reagent (Thermo Scientific: A34807) was resuspended into anhydrous acetonitrile. In total, 30 µL of the corresponding reagent was added to the samples and 60 µL to the pools, as shown in Table 1 and Table 2, and the reaction was incubated at room temperature for one hour.

The reaction was quenched with 5 µL 5% hydroxylamine (Sigma-Aldrich: 159417). Samples from run 1, in addition to half of pool 1 and pool 2 each, were then pooled to form tube 1, and samples from run 2 were then pooled with the remaining pool 1 and pool 2 to form tube 2. The combined samples were then diluted 1:15 times with 0.1% TFA, then the solid-phase extraction was repeated to desalt the combined samples.

#### 4.4.4. Reverse-Phase Liquid Chromatography

Samples from tubes 1 and 2 were suspended in Buffer A (95% water, 5% acetonitrile, 10mM NH_4_HCO_3_, pH 9.0). These samples were then separated by reverse-phase liquid chromatography with an ÄKTA pure (GE Healthcare Life Sciences, MARLBOROUGH, MA, USA) at high pH using a 100 × 4.6 mm Onyx Monolithic C18 column (Phenomenex: CH0-7643, Torrance, CA, USA), with a flow rate of 1mL/minute. The column was run for 15 min at a gradient of 0–40% of Buffer B (5% water, 95% acetonitrile): Buffer A and then for 5 min up to 100% Buffer B using the protocol in Table 3 below. Sixty fractions of 0.6mL volume were collected, then concatenated into twenty samples by combining fractions 1-21-41, 2-22-42, 3-23-43, etc. [87].

#### 4.4.5. Proteomics Analysis with LC-MS/MS

Analysis of individual fractions was carried out on the Q Exactive HF-X Orbitrap MS instrument (Thermo Fisher Scientific, Bremen, Germany). The sample was introduced using an Easy-nLC 1000 system (Thermo Fisher Scientific) at 1.5 μg per injection. Mobile phase A was 0.1% (v/v) formic acid and mobile phase B was 0.1% (v/v) formic acid in 80% acetonitrile (LC-MS grade). Gradient separation of peptides was performed on a C18 (Luna C18(2), 3 μm particle size (Phenomenex, Torrance, CA, USA)) column packed in-house in Pico-Frit (100 μm × 30 cm) capillaries (New Objective, Woburn, MA, USA). Peptide separation was conducted using the following gradients: starting with 5% of phase B over 2 min, followed by 5–7% over 2 min, 7–25% over 60 min, 25–60% over 15 min, 60–90% over 1 min, with final elution of 90% B for 10 min at a flow rate of 300 nL/min.

Data acquisition on Q Exactive HF-X Orbitrap MS instrument was configured for data-dependent method using the full MS/dd-MS2 setup in a positive mode. Spray voltage was set to 2 kV, funnel RF level at 40, and heated capillary at 275 °C. Survey scans covering the mass range of 350–1500 m/z were acquired at a resolution of 60,000 (at m/z 200), with a maximum ion injection time of 60 milliseconds and a normalized automatic gain control (AGC) target set to 3e6. This was followed by MS2 acquisition at a resolution of 45,000; selected ions were fragmented at 32% normalized collision energy, with intensity threshold kept at 5.8e4. AGC target value for fragment spectra was set to 1e5, with a maximum ion injection time set to 86ms and an isolation width set at 0.7 m/z. Dynamic exclusion of previously selected masses was enabled for 20.0 s, charge-state filtering was limited to 2–6, peptide match was set to preferred, and isotope exclusion was on.

Raw MS data were analyzed using Proteome Discoverer 2.2 (ThermoFisher Scientific). Peak lists were searched against the UniProt database as well as the cRAP database of common contaminants (Global Proteome Machine Organization). Cysteine carbamidomethylation was set as a fixed modification, while methionine oxidation, N-terminal methionine loss, and phosphorylation on serine, threonine, and tyrosine were included as variable modifications. A mass accuracy tolerance of 5 ppm was used for precursor ions, while 0.02 Da was used for HCD fragmentation for product ions. Database search was performed using SequestHT with Percolator validation, which used both forward and reverse databases to score the peptide-spectrum matches (PSMs) and determine the false discovery rates (FDRs) [88]. Percolator was used to determine confident peptide identifications using a 0.1% false discovery rate (FDR). All unique and razor peptides were used for quantification, and the number of peptides used for each protein after statistical analysis (in Section 4.6 below) is shown in Table S7.

### 4.5. Proteomic Data Analysis

The protein identification and quantification result files generated by Proteome Discoverer 2.2 were then simplified using regular expressions in Python 3.7 to extract accession ID and abundances for each sample. The resulting set of 2708 individual proteins (Table S8) detected in all conditions was used for further analysis. Gene ontology (GO) tags were taken from the “UniProt” database to identify the biological processes associated with these proteins.

To assess the reproducibility of the abundances, a simple bivariate correlation experiment for the first twenty proteins identified was then performed. For each mass spectrometry experimental run, the correlation between the abundance values per protein for all sample replicates and the abundance values per protein for the pool replicates was determined. The resulting means and Pearson correlation coefficients for each protein per run were added to Tables S1 and S2.

To enable comparisons between two multithreaded LC-MS/MS experiments, the scaling factor described by Plubell et al. [89] was employed to scale all abundances by the geometric mean of all pools used for the TMT experiment.

After a datasheet was produced with the scaling factors for each accession ID, the abundances for each protein in each experimental condition were then multiplied by the scaling factors to normalize between both multithreaded experiments. Additionally, each step for automation included manual validation.

### 4.6. Statistical Analysis

Estimates of the relative abundance of proteins were analyzed using a 2-way analysis of variance (ANOVA) for growth media (AD, OM, SM) and temperature (37 °C, 42 °C) as shown in Table 4. Where significant interactions were observed, Duncan’s multiple range post hoc tests (*p* < 0.05) were used to identify the influence of temperature and growth media that contributed to the observed significant differences [90]. In total, 81 proteins were shown to have altered levels in the interaction between temperature and growth media (Tables S9–S89). For the full-rank model ANOVAs, each population for a given temperature and growth media had the same number of samples (n = 3); therefore, the means were comparable and did not need to be weighted. For proteins without all samples in each condition (i.e., n < 3), ANOVAs were still performed, but the results from these ANOVAs were labelled as non-full-rank model. 

Whereas SAS software, version 9.4, was initially used to evaluate relative abundance, all ANOVAs were validated by an independent operator using Python 3.7. 

## 5. Conclusions

Future research is necessary to determine whether the changes in protein levels were at either an epigenetic (DNA methylation) or a genetic level (DNA mutation). If these changes occurred at an epigenetic level, the changes to the proteomic profiles of the differentiated cells could be more prolonged and potentially result in effects during embryonic or childhood development and may even persist into adulthood. In the present study, heat stress was selected for simplicity and as a potential result of climate stress, but other stresses could be applied to the cells, such as chemical-induced stress or physical stresses. For example, proinflammatory cytokines such as IL-6 or TNF-α could be added to the media during the differentiation period, yielding greater detail about the specificity of the proteomic changes based on different stresses. Human cell lines are an obvious next step, as they are useful for confirming whether the model is generalizable to human cells or specific to equine cells. 

In the present study, all cells were harvested on day 10 of the final passage, so that bias due to collection time was minimized in the experiment. However, a possible next step could be to consider a time series of protein expression by the cells. The advantage of a time series, such as at 3, 5, 7, and 10 days after differentiation, would be to investigate the effect on the timeline of differentiation, as some proteins may be more upregulated during the initial processes, and then disappear over time in mature tissue cells.

Finally, this study represents a first step in demonstrating the potential epigenetic effects of climate change, specifically heat stress on development by comparing protein levels for two specific cell types versus a control. The next steps in this research program require the investigation of the causal mechanisms that are responsible for the differences in the observed protein levels.

## Figures and Tables

**Table 1:** LC-MS/MS experiment 1. SM is protein samples from cells grown in standard media, OM from cells grown in osteogenic media, and AD from cells grown in adipogenic media.

Sample No.	Condition	Reagent
S1	SM1 37 °C	TMT^10^-126
S2	SM2 37 °C	TMT^10^-127N
S3	SM3 37 °C	TMT^10^-127C
S4	OM1 37 °C	TMT^10^-128N
S5	OM2 37 °C	TMT^10^-128C
S6	OM3 37 °C	TMT^10^-129N
S7	AD1 37 °C	TMT^10^-129C
S8	AD2 37 °C	TMT^10^-130N
S9	AD3 37 °C	TMT^10^-130C
Pool 1	All	TMT^10^-131
Pool 2	All	TMT^11^-131C

**Table 2:** LC-MS/MS experiment 2. SM is protein samples from cells grown in standard media, OM from cells grown in osteogenic media, and AD from cells grown in adipogenic media.

Sample No.	Condition	Reagent
S10	SM1 42 °C	TMT^10^-126
S11	SM2 42 °C	TMT^10^-127N
S12	SM3 42 °C	TMT^10^-127C
S13	OM1 42 °C	TMT^10^-128N
S14	OM2 42 °C	TMT^10^-128C
S15	OM3 42 °C	TMT^10^-129N
S16	AD1 42 °C	TMT^10^-129C
S17	AD2 42 °C	TMT^10^-130N
S18	AD3 42 °C	TMT^10^-130C
Pool 1	All	TMT^10^-131
Pool 2	All	TMT^11^-131C

**Table 3:** Protocol for reverse-phase liquid chromatography.

Step	CV (Column Volumes)
Equilibrate	0.83
Sample application	0.5
Column wash	0.83
Elution	16
Column wash	0.83
Equilibrate	1.66

**Table 4:** Matrix for two-way ANOVA.

SM	OM	AD	
${\overset{¯}{\underset{}{x}}}_{13}$ n = 3	${\overset{¯}{\underset{}{x}}}_{12}$ n = 3	${\overset{¯}{\underset{}{x}}}_{11}$ n = 3	37 °C
${\overset{¯}{\underset{}{x}}}_{23}$ n = 3	${\overset{¯}{\underset{}{x}}}_{23}$ n = 3	${\overset{¯}{\underset{}{x}}}_{21}$ n = 3	42 °C

## Data Availability

The mass spectrometry proteomics data were deposited into the ProteomeXchange Consortium via the PRIDE [91] partner repository with the dataset identifier PXD033414.

## Supplementary Materials

The following supporting information can be downloaded at: https://www.mdpi.com/article/10.3390/ijms23137233/s1.
