# Supplementary material for: Heat Shock Alters the Proteomic Profile of Equine Mesenchymal Stem Cells
Source: Int J Mol Sci. 2022 Jun 29;23(13):7233. doi: 10.3390/ijms23137233 (PMC9267023; doi:10.3390/ijms23137233)
Supplement: Supplementary file 1 [file ijms-23-07233-s001.zip › ijms-1729675-supplementary/Table S7 Unique and Razor peptides.pdf]

Table S7. Unique and razor peptides used for protein quantification by SequestHT.

Accession Number	Gene names	Unique and razor peptides
A0A3Q2H363	DPYSL2	4
A0A3Q2HX46	SRSF7	7
A0A3Q2H9C5	DDX17	17
A0A3Q2KZM7	SEC23A	7
A0A5F5PVR8	ROR2	5
A0A3Q2HGX2	PPT1	3
A0A3Q2LU22	YME1L1	4
F7CFK9	SDF2	2
F6RG06	COG8	3
F6XF99	CD58	1
F7B5C4	VIM	65
F7BNQ8	ENTPD5	2
A0A3Q2I292	TTC17 API5	5
F7CQ91	COA3	3
A0A3Q2I2V4	TRIM24	1
F6TYR9	DSE	3
F6ULU1	TST	2
A0A5F5PZG0	COL11A1	10
F6YP32	DPP7	3
F7CLX6	CHP1	3
F6YUS5	DAAM2	1
A0A3Q2IB34	PICALM	7
A0A3Q2HLL1	AKNA	1
A0A5F5PRZ1	RPL37	5
F7C5Z0	HMGA1	4
F6VAP5	RAB4B	3
F6ZB28	PBXIP1	2
F6SUF8	C7H19orf53	3
A0A3Q2HJC4	SERINC1	1
A0A3Q2HRV7		4
A0A3Q2KUB6	GXYLT1	1
F7C3B4	XPO7	2

A0A3Q2IEL7	SPECC1	6
F6SLU7	C5H1orf21	3
F7BK32	POGLUT2	2
A0A3Q2HPI7	SH3PXD2B	4
F6QCC7	LOC100033941	4
A0A3Q2II24	RBPJ	5
F7B5A3	FYTTD1	4
F6PVJ6	OGN	2
K9KA63		2
A0A3Q2HGR5	VPS45<br>PLEKHO1	2
F6T767	PDIA5	13
A0A5F5PXG1	FDPS	2
A0A3Q2I2M2	SEC13	2
A0A5F5Q219	MED14	4
F6TYZ0	TFCP2	2
F6ZPY1	EFHD2	2
F7D5H0	SNX18	3
F6W019	SFSWAP	2
F6T1P0	PTGR2	1
F6YTB8	BMP1	4
A0A3Q2HG96	ADAM17	4
A0A3Q2H452	SEPTIN9	9
A0A3Q2ICA0	PLS3	17
A0A3Q2I9F2		6
A0A5F5Q3Z6	UBA5	3
A0A3Q2HP57	IGF2R	8
F6TZL2	U2AF1	8
A0A5F5PNC6	SERPINB10<br>SERPINB2	2
F6REC8	BCKDHA	4
F6ZNX3	COL14A1	18
F7CL80	DSTN	6
F7BXA6	NPEPPS	4
F7CAN3	LRRCC1	1
F7C4W2	OSGEP	1

F7APL8	ZRANB2	1
A0A3Q2HEJ2	TOMM40	4
A0A3Q2I4J6	HIP1	7
F6TMS0	SEL1L	2
F6XL78	APP	9
F6UJZ8	PRCP	3
F7DBF8	VAMP3	3
A0A5F5PF14	BASP1	3
H9GZW3	SGTA	3
A0A3Q2LA78	MFF	2
A0A3Q2KJ26	CYB5B	1
F7DBU9	EIF4E2	1
A0A3Q2LT78	BICC1	1
A0A3Q2H8V2	RRP36	1
F7CE39	UNC93B1	1
