# Supplementary material for: Heat Shock Alters the Proteomic Profile of Equine Mesenchymal Stem Cells
Source: Int J Mol Sci. 2022 Jun 29;23(13):7233. doi: 10.3390/ijms23137233 (PMC9267023; doi:10.3390/ijms23137233)
Supplement: Supplementary file 1 [file ijms-23-07233-s001.zip › ijms-1729675-supplementary/Tables S1 and S2 Correlation coefficients.pdf]

Table S1. Correlation coefficients for triplicates of twenty proteins and pooled samples from LC-MS Experiment 1.

Protein	Triplicate Abundance	Pooled Abundance	Correlation Coefficient
A0A3Q2H363	2623657 ± 286561	2753633 ± 30277	1.00000
A0A3Q2HX46	16740198 ± 2908517	15500797 ± 320907	1.00000
A0A3Q2H9C5	43487812 ± 9390661	44476732 ± 1032070	1.00000
A0A3Q2KZM7	16339474 ± 5052380	15211336 ± 276623	-1.00000
A0A5F5PVR8	884488 ± 237613	852610 ± 82269	1.00000
A0A3Q2HGX2	4317874 ± 1387174	4231959 ± 524763	-1.00000
A0A3Q2LU22	15204903 ± 3325296	15474919 ± 552172	-1.00000
F7CFK9	724208 ± 91118	748525 ± 8212	1.00000
F6RG06	608874 ± 134060	581997 ± 11736	-1.00000
F6XF99	487339 ± 96617	466396 ± 38343	-1.00000
F7B5C4	4480575546 ± 967489441	5036961288 ± 76797930	-1.00000
F7BNQ8	175239 ± 28859	175864 ± 27502	-1.00000
A0A3Q2I292	6449322 ± 942442	6164120 ± 73940	1.00000
F7CQ91	4349976 ± 941576	4359296 ± 292325	-1.00000
A0A3Q2I2V4	950634 ± 234756	1739901 ± 943056	1.00000
F6TYR9	2271819 ± 853758	2229100 ± 157253	1.00000
F6ULU1	3295372 ± 452019	3248867 ± 79145	1.00000
A0A5F5PZG0	6903302 ± 4956199	5036747 ± 464656	-1.00000
F6YP32	1463209 ± 277213	1470296 ± 179532	-1.00000
F7CLX6	2014998 ± 279177	2000850 ± 34730	-1.00000

Table S2. Correlation coefficients for triplicates of twenty proteins and pooled samples from LC-MS Experiment 2.

Protein	Triplicate Abundance	Pooled Abundance	Correlation Coefficient
A0A3Q2H363	306052 ± 53306	307773 ± 74858	1.00000
A0A3Q2HX46	29948807 ± 6239874	26538981 ± 648169	1.00000
A0A3Q2H9C5	30666573 ± 8904900	31449851 ± 1315671	1.00000
A0A3Q2KZM7	3147833 ± 1114496	3131992 ± 83251	1.00000
A0A5F5PVR8	1162999 ± 221485	1088484 ± 289221	-1.00000
A0A3Q2HGX2	5912794 ± 1056625	5483164 ± 709332	1.00000
A0A3Q2LU22	13164804 ± 3140733	14231602 ± 1431512	1.00000
F7CFK9	1756610 ± 325042	1613455 ± 104281	1.00000
F6RG06	772296 ± 161892	668429 ± 15871	-1.00000
F6XF99	340523 ± 76044	289037 ± 2959	-1.00000
F7B5C4	5912621780 ± 2342701477	5762520326 ± 24674860	-1.00000
F7BNQ8	639800 ± 97661	592821 ± 89370	-1.00000
A0A3Q2I292	14209025 ± 1458210	14643897 ± 232645	-1.00000
F7CQ91	10160197 ± 1938326	11005433 ± 257024	1.00000
A0A3Q2I2V4	1301579 ± 240582	2097823 ± 1276880	-1.00000
F6TYR9	282697 ± 53084	257275 ± 6221	1.00000
F6ULU1	3075607 ± 643169	3232296 ± 134800	-1.00000
A0A5F5PZG0	99310 ± 39937	64032 ± 7348	-1.00000
F6YP32	218844 ± 83824	212902 ± 9277	1.00000
F7CLX6	707785 ± 54293	715906 ± 69911	-1.00000
