# Supplementary material for: Heat Shock Alters the Proteomic Profile of Equine Mesenchymal Stem Cells
Source: Int J Mol Sci. 2022 Jun 29;23(13):7233. doi: 10.3390/ijms23137233 (PMC9267023; doi:10.3390/ijms23137233)
Supplement: Supplementary file 1 [file ijms-23-07233-s001.zip › ijms-1729675-supplementary/Tables S3-S6 Gene names and descriptions.pdf]

Table S3. Proteins with altered protein levels between 37°C and 42°C for osteoblasts.

Protein	Protein names	Gene names
A0A3Q2HX46	Serine and arginine rich splicing factor 7	SRSF7
A0A3Q2H9C5	RNA helicase (EC 3.6.4.13)	DDX17
A0A5F5PVR8	Receptor tyrosine kinase like orphan receptor 2	ROR2
A0A3Q2HGX2	Palmitoyl-protein hydrolase 1 (EC 3.1.2.22) (Palmitoyl-protein thioesterase 1)	PPT1
F6RG06	Conserved oligomeric Golgi complex subunit 8 (COG complex subunit 8) (Component of oligomeric Golgi complex 8)	COG8
F7BNQ8	Ectonucleoside triphosphate diphosphohydrolase 5 (inactive)	ENTPD5
F6TYR9	Dermatan sulfate epimerase	DSE
F6YP32	Dipeptidyl peptidase 7	DPP7
F6YUS5	Dishevelled associated activator of morphogenesis 2	DAAM2
A0A3Q2HRV7	Profilin	
A0A3Q2KUB6	Glucoside xylosyltransferase 1	GXYLT1
A0A3Q2IEL7	Sperm antigen with calponin homology and coiled-coil domains 1	SPECC1
F7BK32	Protein O-glucosyltransferase 2	POGLUT2
A0A3Q2II24	Recombination signal binding protein for immunoglobulin kappa J region	RBPJ
A0A3Q2HGR5	Vacuolar protein sorting 45 homolog	VPS45
F6W019	Splicing factor SWAP	SFSWAP
F7CL80	Actin-depolymerizing factor (Destrin)	DSTN
F7BXA6	Aminopeptidase (EC 3.4.11.-)	NPEPPS
F7B5C4	Vimentin	VIM
F7CLX6	Calcineurin like EF-hand protein 1	CHP1
A0A3Q2HG96	ADAM metallopeptidase domain 17	ADAM17
**Incomplete sets**		
F6XL78	ABPP (Alpha-secretase C-terminal fragment) (Alzheimer disease amyloid A4 protein homolog) (Amyloid precursor protein) (Amyloid-beta A4 protein) (Amyloid-beta precursor protein) (Amyloid-beta protein 40) (Amyloid-beta protein 42) (Beta-APP40) (Beta-APP42) (Beta-secretase C-terminal fragment) (C31) (C80) (C83) (C99) (Gamma-CTF(50)) (Gamma-CTF(57)) (Gamma-CTF(59)) (Gamma-secretase C-terminal fragment 50) (Gamma-secretase C-terminal fragment 57) (Gamma-secretase C-terminal fragment 59) (N-APP) (P3(40)) (P3(42)) (Soluble APP-alpha) (Soluble APP-beta)	APP

Table S4. Proteins with increased protein levels between 37°C and 42°C for osteoblasts.

Protein	Protein names	Gene names
A0A3Q2HX46	Serine and arginine rich splicing factor 7	SRSF7
A0A3Q2H9C5	RNA helicase (EC 3.6.4.13)	DDX17
A0A5F5PVR8	Receptor tyrosine kinase like orphan receptor 2	ROR2
A0A3Q2HGX2	Palmitoyl-protein hydrolase 1 (EC 3.1.2.22) (Palmitoyl-protein thioesterase 1)	PPT1
F6RG06	Conserved oligomeric Golgi complex subunit 8 (COG complex subunit 8) (Component of oligomeric Golgi complex 8)	COG8
F7BNQ8	Ectonucleoside triphosphate diphosphohydrolase 5 (inactive)	ENTPD5
F6TYR9	Dermatan sulfate epimerase	DSE
F6YP32	Dipeptidyl peptidase 7	DPP7
F6YUS5	Dishevelled associated activator of morphogenesis 2	DAAM2
A0A3Q2HRV7	Profilin	
A0A3Q2KUB6	Glucoside xylosyltransferase 1	GXYLT1
A0A3Q2IEL7	Sperm antigen with calponin homology and coiled-coil domains 1	SPECC1
F7BK32	Protein O-glucosyltransferase 2	POGLUT2
A0A3Q2II24	Recombination signal binding protein for immunoglobulin kappa J region	RBPJ
A0A3Q2HGR5	Vacuolar protein sorting 45 homolog	VPS45
F6W019	Splicing factor SWAP	SFSWAP
F7CL80	Actin-depolymerizing factor (Destrin)	DSTN
F7BXA6	Aminopeptidase (EC 3.4.11.-)	NPEPPS

Table S5. Proteins with decreased protein levels between 37°C and 42°C for osteoblasts.

Protein	Protein names	Gene names
F7B5C4	Vimentin	VIM
F7CLX6	Calcineurin like EF-hand protein 1	CHP1
A0A3Q2HG96	ADAM metallopeptidase domain 17	ADAM17
**Incomplete sets**		
F6XL78	ABPP (Alpha-secretase C-terminal fragment) (Alzheimer disease amyloid A4 protein homolog) (Amyloid precursor protein) (Amyloid-beta A4 protein) (Amyloid-beta precursor protein) (Amyloid-beta protein 40) (Amyloid-beta protein 42) (Beta-APP40) (Beta-APP42) (Beta-secretase C-terminal fragment) (C31) (C80) (C83) (C99) (Gamma-CTF(50)) (Gamma-CTF(57)) (Gamma-	APP

	CTF(59)) (Gamma-secretase C-terminal fragment 50) (Gamma-secretase C-terminal fragment 57) (Gamma-secretase C-terminal fragment 59) (N-APP) (P3(40)) (P3(42)) (Soluble APP-alpha) (Soluble APP-beta)	

Table S6. Proteins with altered protein levels between 37°C and 42°C for adipocytes.

Protein	Protein names	Gene names
F7B5C4	Vimentin	VIM
A0A3Q2H363	Dihydropyrimidinase-related protein 2	DPYSL2
A0A3Q2I292	Tetratricopeptide repeat domain 17	TTC17
F6VAP5	Ras-related protein Rab-4	RAB4B
A0A3Q2II24	Recombination signal binding protein for immunoglobulin kappa J region	RBPJ
F7D5H0	Sorting nexin	SNX18
F6W019	Splicing factor SWAP	SFSWAP
A0A5F5Q3Z6	UFM1-activating enzyme (Ubiquitin-like modifier-activating enzyme 5)	UBA5
F6TZL2	Uncharacterized protein	U2AF1
A0A3Q2I4J6	Huntingtin interacting protein 1	HIP1
F6TMS0	SEL1L adaptor subunit of ERAD E3 ubiquitin ligase (Suppressor of lin-12-like protein)	SEL1L
**Incomplete sets**		
F6XL78	ABPP (Alpha-secretase C-terminal fragment) (Alzheimer disease amyloid A4 protein homolog) (Amyloid precursor protein) (Amyloid-beta A4 protein) (Amyloid-beta precursor protein) (Amyloid-beta protein 40) (Amyloid-beta protein 42) (Beta-APP40) (Beta-APP42) (Beta-secretase C-terminal fragment) (C31) (C80) (C83) (C99) (Gamma-CTF(50)) (Gamma-CTF(57)) (Gamma-CTF(59)) (Gamma-secretase C-terminal fragment 50) (Gamma-secretase C-terminal fragment 57) (Gamma-secretase C-terminal fragment 59) (N-APP) (P3(40)) (P3(42)) (Soluble APP-alpha) (Soluble APP-beta)	APP
F7DBF8	Vesicle associated membrane protein 3	VAMP3
H9GZW3	Small glutamine rich tetratricopeptide repeat containing alpha	SGTA
