# Supplementary material for: Heat Shock Alters the Proteomic Profile of Equine Mesenchymal Stem Cells
Source: Int J Mol Sci. 2022 Jun 29;23(13):7233. doi: 10.3390/ijms23137233 (PMC9267023; doi:10.3390/ijms23137233)
Supplement: Supplementary file 1 [file ijms-23-07233-s001.zip › ijms-1729675-supplementary/Tables S9-S89 Duncan Summary Tables.pdf]

Data in the following section refers to comparison of individual mean abundance values per protein analyzed by 2-way ANOVA using Temperature (37 vs 42) and Growth Media (SM vs OM vs AD)

Table S9: Uniprot accession number=A0A3Q2H363

Means with the same letter are not significantly different.				
Duncan Grouping		Mean	N	GRP
	A	1063165	3	SM_42
	A			
B	A	952060	3	AD_37
B	A			
B	A	939287	3	OM_42
B				
B	C	902304	3	OM_37
	C			
D	C	777059	3	SM_37
D				
D		743884	3	AD_42

Table S10: Uniprot accession number=A0A3Q2HX46

Means with the same letter are not significantly different.				
Duncan Grouping		Mean	N	GRP
	A	27824728	3	OM_42
	A			
B	A	23942808	3	AD_37
B	A			
B	A	23220239	3	SM_37
B	A			
B	A	21872084	3	SM_42
B				
B		18968229	3	AD_42
B				
B		18549297	3	OM_37

Table S11: Uniprot accession number=A0A3Q2H9C5

Means with the same letter are not significantly different.				
Duncan Grouping		Mean	N	GRP
	A	49281570	3	OM_42
	A			
B	A	40657609	3	SM_37
B				
B		35785861	3	AD_37
B				
B		33262856	3	OM_37
B				
B		31199908	3	SM_42

Means with the same letter are not significantly different.				
Duncan Grouping		Mean	N	GRP
B				
B		28925149	3	AD_42

Table S12: Uniprot accession number=A0A3Q2KZM7

Means with the same letter are not significantly different.				
Duncan Grouping		Mean	N	GRP
	A	9400483	3	OM_42
	A			
	A	9317143	3	SM_37
	A			
B	A	6765703	3	AD_37
B	A			
B	A	6476142	3	SM_42
B	A			
B	A	6159774	3	OM_37
B				
B		4935013	3	AD_42

Table S13: Uniprot accession number=A0A5F5PVR8

Means with the same letter are not significantly different.				
Duncan Grouping		Mean	N	GRP
	A	1262057	3	SM_37
	A			
	A	1183382	3	OM_42
	A			
B	A	986054	3	AD_42
B	A			
B	A	985621	3	AD_37
B	A			
B	A	918475	3	SM_42
B				
B		750441	3	OM_37

Table S14: Uniprot accession number=A0A3Q2HGX2

Means with the same letter are not significantly different.				
Duncan Grouping		Mean	N	GRP
	A	6127298	3	SM_37
	A			
	A	6039437	3	OM_42
	A			
	A	5224601	3	AD_37
	A			

Means with the same letter are not significantly different.				
Duncan Grouping		Mean	N	GRP
	A	5218990	3	AD_42
	A			
B	A	4325220	3	SM_42
B				
B		3392809	3	OM_37

Table S15: Uniprot accession number=A0A3Q2LU22

Means with the same letter are not significantly different.				
Duncan Grouping		Mean	N	GRP
	A	17850546	3	AD_42
	A			
B	A	15960029	3	SM_37
B	A			
B	A	14127413	3	OM_37
B	A			
B	A	13656470	3	AD_37
B				
B		12204466	3	SM_42
B				
B		11128458	3	OM_42

Table S16: Uniprot accession number=F7CFK9

Means with the same letter are not significantly different.					
Duncan Grouping			Mean	N	GRP
	A		1354976	3	SM_42
	A				
B	A		1273413	3	AD_42
B	A				
B	A	C	1123137	3	OM_37
B		C			
B		C	1078725	3	AD_37
		C			
		C	987913	3	SM_37
		C			
		C	961005	3	OM_42

Table S17: Uniprot accession number=F6RG06

Means with the same letter are not significantly different.				
Duncan Grouping		Mean	N	GRP
	A	866535	3	OM_42
	A			
B	A	728213	3	SM_37

Means with the same letter are not significantly different.				
Duncan Grouping		Mean	N	GRP
B	A			
B	A	696729	3	AD_37
B	A			
B	A	651491	3	SM_42
B	A			
B	A	643885	3	AD_42
B				
B		532621	3	OM_37

Table S18: Uniprot accession number=F6XF99

Means with the same letter are not significantly different.				
Duncan Grouping		Mean	N	GRP
	A	532813	3	OM_42
	A			
B	A	458152	3	AD_37
B				
B	C	391322	3	SM_42
B	C			
B	C	374705	3	SM_37
B	C			
B	C	373548	3	AD_42
	C			
	C	318080	3	OM_37

Table S19: Uniprot accession number=F7B5C4

Means with the same letter are not significantly different.					
Duncan Grouping			Mean	N	GRP
	A		6808321187	3	AD_42
	A				
	A		6737250900	3	SM_42
	A				
B	A		5454361326	3	OM_37
B	A				
B	A	C	4590112220	3	SM_37
B		C			
B		C	4332814954	3	AD_37
		C			
		C	3038051244	3	OM_42

Table S20: Uniprot accession number=F7BNQ8

Means with the same letter are not significantly different.				
Duncan Grouping		Mean	N	GRP
	A	402287	3	OM_42
	A			
B	A	383418	3	AD_37
B				
B	C	323653	3	AD_42
B	C			
B	C	319485	3	SM_42
	C			
	C	293893	3	SM_37
	C			
	C	287907	3	OM_37

Table S21: Uniprot accession number=A0A3Q2I292

Means with the same letter are not significantly different.				
Duncan Grouping		Mean	N	GRP
	A	10585133	3	AD_37
	A			
	A	10337380	3	SM_37
	A			
B	A	9915980	3	OM_42
B	A			
B	A	9610816	3	SM_42
B	A			
B	A	8898892	3	OM_37
B				
B		8129420	3	AD_42

Table S22: Uniprot accession number=F7CQ91

Means with the same letter are not significantly different.				
Duncan Grouping		Mean	N	GRP
	A	8611555	3	OM_37
	B	7097107	3	AD_42
	B			
	B	7068554	3	SM_42
	B			
C	B	6595144	3	AD_37
C				
C	D	5528275	3	SM_37
	D			
	D	5017845	3	OM_42

Table S23: Uniprot accession number=A0A3Q2I2V4

Means with the same letter are not significantly different.				
Duncan Grouping		Mean	N	GRP
	A	1336229	3	SM_42
	A			
	A	1242205	3	OM_42
	A			
B	A	1163950	3	SM_37
B	A			
B	A	1163792	3	AD_37
B	A			
B	A	977631	3	AD_42
B				
B		803789	3	OM_37

Table S24: Uniprot accession number=F6TYR9

Means with the same letter are not significantly different.				
Duncan Grouping		Mean	N	GRP
	A	1069723	3	AD_37
	A			
	A	872439	3	OM_42
	A			
B	A	854902	3	SM_42
B	A			
B	A	769024	3	AD_42
B	A			
B	A	725026	3	SM_37
B				
B		520667	3	OM_37

Table S25: Uniprot accession number=F6ULU1

Means with the same letter are not significantly different.					
Duncan Grouping			Mean	N	GRP
	A		3760668	3	AD_42
	A				
B	A		3692205	3	OM_37
B	A				
B	A	C	3093221	3	SM_37
B	A	C			
B	A	C	3075446	3	AD_37
B		C			
B		C	2945987	3	SM_42
		C			
		C	2543786	3	OM_42

Table S26: Uniprot accession number=A0A5F5PZG0

Means with the same letter are not significantly different.				
Duncan Grouping		Mean	N	GRP
	A	1430965	3	SM_37
	A			
B	A	993322	3	OM_42
B	A			
B	A	975361	3	AD_42
B				
B		673666	3	SM_42
B				
B		541634	3	AD_37
B				
B		362481	3	OM_37

Table S27: Uniprot accession number=F6YP32

Means with the same letter are not significantly different.				
Duncan Grouping		Mean	N	GRP
	A	824351	3	OM_42
	A			
B	A	633553	3	SM_37
B				
B	C	575255	3	AD_37
B	C			
B	C	491801	3	AD_42
B	C			
B	C	461571	3	OM_37
	C			
	C	409167	3	SM_42

Table S28: Uniprot accession number=F7CLX6

Means with the same letter are not significantly different.				
Duncan Grouping		Mean	N	GRP
	A	1387390	3	OM_37
	A			
B	A	1232185	3	SM_42
B				
B		1159030	3	OM_42
B				
B		1158569	3	AD_42
B				
B		1152739	3	SM_37
B				
B		1075774	3	AD_37

Table S29: Uniprot accession number=F6YUS5

Means with the same letter are not significantly different.				
Duncan Grouping		Mean	N	GRP
	A	970816	3	OM_42
	A			
B	A	866745	3	SM_37
B	A			
B	A	828788	3	SM_42
B	A			
B	A	821258	3	AD_37
B	A			
B	A	727154	3	AD_42
B				
B		649291	3	OM_37

Table S30: Uniprot accession number=A0A3Q2IB34

Means with the same letter are not significantly different.				
Duncan Grouping		Mean	N	GRP
	A	9005892	3	SM_37
	A			
B	A	7156458	3	OM_42
B	A			
B	A	6983995	3	AD_37
B				
B		6537150	3	SM_42
B				
B		5628115	3	AD_42
B				
B		5019393	3	OM_37

Table S31: Uniprot accession number=A0A3Q2HLL1

Means with the same letter are not significantly different.					
Duncan Grouping			Mean	N	GRP
	A		516384	3	AD_37
	A				
B	A		490681	3	OM_42
B	A				
B	A	C	392277	3	OM_37
B		C			
B		C	353173	3	SM_37
		C			
		C	325433	3	SM_42

Means with the same letter are not significantly different.					
Duncan Grouping			Mean	N	GRP
		C			
		C	297523	3	AD_42

Table S32: Uniprot accession number=A0A5F5PRZ1

Means with the same letter are not significantly different.					
Duncan Grouping			Mean	N	GRP
	A		6891021	3	OM_37
	A				
B	A		6211952	3	SM_42
B	A				
B	A	C	4643550	3	SM_37
B		C			
B		C	4382283	3	OM_42
B		C			
B		C	4263120	3	AD_37
		C			
		C	3727206	3	AD_42

Table S33: Uniprot accession number=F7C5Z0

Means with the same letter are not significantly different.			
Duncan Grouping	Mean	N	GRP
A	8022521	3	OM_37
B	4458992	3	AD_37
B			
B	4347790	3	SM_42
B			
B	4004193	3	AD_42
B			
B	2099477	3	OM_42
B			
B	2079969	3	SM_37

Table S34: Uniprot accession number=F6VAP5

Means with the same letter are not significantly different.				
Duncan Grouping		Mean	N	GRP
	A	1191738	3	SM_42
	A			
	A	1151519	3	AD_37
	A			
B	A	1094228	3	OM_42
B	A			

Means with the same letter are not significantly different.				
Duncan Grouping		Mean	N	GRP
B	A	1043146	3	SM_37
B	A			
B	A	1038571	3	OM_37
B				
B		924651	3	AD_42

Table S35: Uniprot accession number=F6ZB28

Means with the same letter are not significantly different.			
Duncan Grouping	Mean	N	GRP
A	900106	3	OM_42
A			
A	791354	3	AD_37
A			
A	730501	3	SM_42
A			
A	725888	3	SM_37
A			
A	719799	3	AD_42
B	507181	3	OM_37

Table S36: Uniprot accession number=F6SUF8

Means with the same letter are not significantly different.					
Duncan Grouping			Mean	N	GRP
	A		2372817	3	OM_37
	A				
B	A		2099941	3	AD_42
B	A				
B	A	C	1970945	3	AD_37
B		C			
B		C	1727296	3	SM_42
B		C			
B		C	1659230	3	SM_37
		C			
		C	1477548	3	OM_42

Table S37: Uniprot accession number=A0A3Q2HJC4

Means with the same letter are not significantly different.				
Duncan Grouping		Mean	N	GRP
	A	283979	3	SM_42
	A			
	A	267574	3	OM_37

Means with the same letter are not significantly different.				
Duncan Grouping		Mean	N	GRP
	A			
	A	267263	3	AD_37
	A			
B	A	241433	3	AD_42
B	A			
B	A	218970	3	SM_37
B				
B		192607	3	OM_42

Table S38: Uniprot accession number=A0A3Q2HRV7

Means with the same letter are not significantly different.			
Duncan Grouping	Mean	N	GRP
A	14609457	3	OM_42
B	11367389	3	AD_37
B			
B	10955761	3	SM_37
B			
B	10954852	3	SM_42
B			
B	10949822	3	OM_37
B			
B	9872883	3	AD_42

Table S39: Uniprot accession number=A0A3Q2KUB6

Means with the same letter are not significantly different.				
Duncan Grouping		Mean	N	GRP
	A	617693	3	OM_42
	B	421840	3	SM_37
	B			
	B	419145	3	AD_37
	B			
C	B	362717	3	AD_42
C				
C		230720	3	OM_37
C				
C		224447	3	SM_42

Table S40: Uniprot accession number=F7C3B4

Means with the same letter are not significantly different.				
Duncan Grouping		Mean	N	GRP
	A	282451	3	OM_42
	A			
B	A	197790	3	AD_37
B				
B		179771	3	SM_37
B				
B		174079	3	OM_37
B				
B		152130	3	AD_42
B				
B		134524	3	SM_42

Table S41: Uniprot accession number=A0A3Q2IEL7

Means with the same letter are not significantly different.				
Duncan Grouping		Mean	N	GRP
	A	1078100	3	AD_37
	A			
	A	1046164	3	SM_37
	A			
	A	1046048	3	OM_42
	A			
	A	917672	3	AD_42
	A			
B	A	861493	3	SM_42
B				
B		686276	3	OM_37

Table S42: Uniprot accession number=F6SLU7

Means with the same letter are not significantly different.			
Duncan Grouping	Mean	N	GRP
A	1328141	3	SM_42
B	953702	3	OM_37
B			
B	947341	3	AD_37
B			
B	740660	3	AD_42
B			
B	724482	3	SM_37
B			
B	721839	3	OM_42

Table S43: Uniprot accession number=F7BK32

Means with the same letter are not significantly different.				
Duncan Grouping		Mean	N	GRP
	A	1689850	3	OM_42
	A			
B	A	1361363	3	AD_37
B	A			
B	A	1298401	3	SM_42
B				
B		1196536	3	SM_37
B				
B		1112343	3	AD_42
B				
B		1031063	3	OM_37

Table S44: Uniprot accession number=A0A3Q2HPI7

Means with the same letter are not significantly different.				
Duncan Grouping		Mean	N	GRP
	A	976084	3	OM_42
	A			
	A	818235	3	SM_37
	A			
B	A	778746	3	AD_37
B				
B	C	540154	3	OM_37
	C			
	C	450734	3	SM_42
	C			
	C	416575	3	AD_42

Table S45: Uniprot accession number=F6QCC7

Means with the same letter are not significantly different.				
Duncan Grouping		Mean	N	GRP
	A	13380968	3	OM_42
	A			
	A	13236323	3	AD_37
	A			
B	A	10911652	3	OM_37
B				
B		10196637	3	SM_42
B				
B		10144310	3	SM_37
B				
B		8918958	3	AD_42

Table S46: Uniprot accession number=A0A3Q2II24

Means with the same letter are not significantly different.					
Duncan Grouping			Mean	N	GRP
	A		2622075	3	OM_42
	A				
B	A		2489049	3	AD_37
B	A				
B	A	C	2123416	3	SM_42
B		C			
B		C	1949679	3	OM_37
		C			
		C	1883854	3	AD_42
		C			
		C	1793059	3	SM_37

Table S47: Uniprot accession number=F7B5A3

Means with the same letter are not significantly different.				
Duncan Grouping		Mean	N	GRP
	A	10468711	3	AD_37
	A			
B	A	9269953	3	OM_37
B	A			
B	A	9230575	3	OM_42
B				
B		8708547	3	SM_42
B				
B		8238910	3	AD_42
B				
B		8128059	3	SM_37

Table S48: Uniprot accession number=F6PVJ6

Means with the same letter are not significantly different.				
Duncan Grouping		Mean	N	GRP
	A	1779555	3	SM_37
	A			
B	A	1280147	3	OM_42
B				
B		825202	3	AD_42
B				
B		821783	3	OM_37
B				
B		769815	3	AD_37
B				
B		744658	3	SM_42

Table S49: Uniprot accession number=K9KA63

Means with the same letter are not significantly different.				
Duncan Grouping		Mean	N	GRP
	A	958589	3	SM_42
	A			
B	A	817558	3	OM_37
B				
B	C	754409	3	AD_37
B	C			
B	C	664359	3	SM_37
	C			
	C	599476	3	AD_42
	C			
	C	574506	3	OM_42

Table S50: Uniprot accession number=A0A3Q2HGR5

Means with the same letter are not significantly different.				
Duncan Grouping		Mean	N	GRP
	A	1100949	3	OM_42
	B	793524	3	SM_37
	B			
C	B	683808	3	AD_37
C	B			
C	B	668003	3	AD_42
C				
C		522342	3	SM_42
C				
C		499142	3	OM_37

Table S51: Uniprot accession number=F6T767

Means with the same letter are not significantly different.				
Duncan Grouping		Mean	N	GRP
	A	21500307	3	SM_37
	A			
	A	20668427	3	AD_37
	A			
	A	19714082	3	OM_42
	A			
B	A	16893630	3	AD_42
B	A			
B	A	16736505	3	SM_42
B				
B		12416336	3	OM_37

Table S52: Uniprot accession number=A0A5F5PXG1

Means with the same letter are not significantly different.				
Duncan Grouping		Mean	N	GRP
	A	2416577	3	SM_37
	A			
B	A	2320052	3	OM_42
B	A			
B	A	2224951	3	AD_42
B	A			
B	A	2048332	3	OM_37
B	A			
B	A	1780242	3	SM_42
B				
B		1693925	3	AD_37

Table S53: Uniprot accession number=A0A3Q2I2M2

Means with the same letter are not significantly different.			
Duncan Grouping	Mean	N	GRP
A	1010008	3	OM_42
B	781322	3	AD_37
B			
B	764329	3	SM_37
B			
B	735033	3	OM_37
B			
B	725994	3	AD_42
B			
B	673107	3	SM_42

Table S54: Uniprot accession number=A0A5F5Q219

Means with the same letter are not significantly different.				
Duncan Grouping		Mean	N	GRP
	A	1618921	3	OM_42
	A			
B	A	1291054	3	AD_37
B				
B		1055900	3	OM_37
B				
B		959624	3	AD_42
B				
B		936550	3	SM_37
B				
B		829805	3	SM_42

Table S55: Uniprot accession number=F6TYZ0

Means with the same letter are not significantly different.				
Duncan Grouping		Mean	N	GRP
	A	780882	3	OM_42
	A			
B	A	669041	3	SM_37
B	A			
B	A	615361	3	AD_37
B	A			
B	A	592863	3	OM_37
B				
B		550465	3	SM_42
B				
B		496463	3	AD_42

Table S56: Uniprot accession number=F6ZPY1

Means with the same letter are not significantly different.				
Duncan Grouping		Mean	N	GRP
	A	1225895	3	SM_42
	A			
B	A	1093717	3	OM_37
B	A			
B	A	1069752	3	OM_42
B	A			
B	A	1035669	3	AD_37
B				
B		935646	3	SM_37
B				
B		934248	3	AD_42

Table S57: Uniprot accession number=F7D5H0

Means with the same letter are not significantly different.				
Duncan Grouping		Mean	N	GRP
	A	1092326	3	OM_42
	A			
	A	1062882	3	SM_37
	A			
	A	979855	3	AD_37
	A			
B	A	883555	3	OM_37
B	A			
B	A	753115	3	SM_42
B				
B		606035	3	AD_42

Table S58: Uniprot accession number=F6W019

Means with the same letter are not significantly different.			
Duncan Grouping	Mean	N	GRP
A	748083	3	OM_42
A			
A	741346	3	AD_37
B	622325	3	SM_42
B			
B	601337	3	AD_42
B			
B	600040	3	SM_37
B			
B	587249	3	OM_37

Table S59: Uniprot accession number=F6T1P0

Means with the same letter are not significantly different.				
Duncan Grouping		Mean	N	GRP
	A	966211	3	OM_42
	A			
B	A	901987	3	AD_37
B				
B	C	773754	3	SM_37
B	C			
B	C	772509	3	OM_37
	C			
	C	716611	3	SM_42
	C			
	C	674320	3	AD_42

Table S60: Uniprot accession number=F6YTB8

Means with the same letter are not significantly different.				
Duncan Grouping		Mean	N	GRP
	A	597574	3	AD_37
	A			
B	A	538898	3	SM_42
B	A			
B	A	397662	3	AD_42
B	A			
B	A	388325	3	OM_42
B				
B		342733	3	SM_37
B				
B		319650	3	OM_37

Table S61: Uniprot accession number=A0A3Q2HG96

Means with the same letter are not significantly different.				
Duncan Grouping		Mean	N	GRP
	A	1185093	3	SM_42
	A			
	A	1174461	3	AD_37
	A			
	A	1167897	3	OM_37
	A			
B	A	967940	3	SM_37
B	A			
B	A	950745	3	AD_42
B				
B		895140	3	OM_42

Table S62: Uniprot accession number=A0A3Q2H452

Means with the same letter are not significantly different.				
Duncan Grouping		Mean	N	GRP
	A	3924958	3	SM_37
	A			
	A	3866467	3	OM_42
	A			
B	A	3411106	3	OM_37
B	A			
B	A	3108285	3	AD_37
B				
B		2769152	3	SM_42
B				
B		2643690	3	AD_42

Table S63: Uniprot accession number=A0A3Q2ICA0

Means with the same letter are not significantly different.				
Duncan Grouping		Mean	N	GRP
	A	45030336	3	OM_42
	A			
	A	43607723	3	SM_37
	A			
B	A	28993099	3	OM_37
B				
B		25470885	3	SM_42
B				
B		24562315	3	AD_37
B				
B		21136085	3	AD_42

Table S64: Uniprot accession number=A0A3Q2I9F2

Means with the same letter are not significantly different.				
Duncan Grouping		Mean	N	GRP
	A	8127365	3	SM_42
	A			
	A	8042289	3	OM_42
	A			
B	A	7467532	3	AD_37
B	A			
B	A	7436315	3	SM_37
B				
B		6021978	3	OM_37
B				
B		5873661	3	AD_42

Table S65: Uniprot accession number=A0A5F5Q3Z6

Means with the same letter are not significantly different.			
Duncan Grouping	Mean	N	GRP
A	1384180	3	AD_37
B	922407	3	SM_42
B			
B	894762	3	OM_37
B			
B	842459	3	OM_42
B			
B	774144	3	SM_37
B			
B	714747	3	AD_42

Table S66: Uniprot accession number=A0A3Q2HP57

Means with the same letter are not significantly different.				
Duncan Grouping		Mean	N	GRP
	A	4446767	3	SM_42
	A			
B	A	4348712	3	OM_37
B	A			
B	A	3408580	3	AD_37
B	A			
B	A	3186398	3	SM_37
B	A			
B	A	3118677	3	OM_42
B				
B		2886388	3	AD_42

Table S67: Uniprot accession number=F6TZL2

Means with the same letter are not significantly different.					
Duncan Grouping			Mean	N	GRP
	A		13428688	3	AD_37
	A				
B	A		11390965	3	OM_42
B	A				
B	A	C	10979709	3	SM_37
B	A	C			
B	A	C	10978902	3	SM_42
B		C			
B		C	8885491	3	OM_37
		C			
		C	8378919	3	AD_42

Table S68: Uniprot accession number=A0A5F5PNC6

Means with the same letter are not significantly different.				
Duncan Grouping		Mean	N	GRP
	A	182604	3	OM_42
	A			
	A	175705	3	AD_37
	A			
B	A	123063	3	AD_42
B	A			
B	A	122591	3	SM_37
B	A			
B	A	109939	3	SM_42
B				
B		59140	3	OM_37

Table S69: Uniprot accession number=F6REC8

Means with the same letter are not significantly different.			
Duncan Grouping	Mean	N	GRP
A	1292280	3	OM_37
A			
A	1239584	3	SM_42
A			
A	1184680	3	AD_37
A			
A	1117829	3	AD_42
A			
A	1084745	3	SM_37

Means with the same letter are not significantly different.			
Duncan Grouping	Mean	N	GRP
B	779707	3	OM_42

Table S70: Uniprot accession number=F6ZNX3

Means with the same letter are not significantly different.			
Duncan Grouping	Mean	N	GRP
A	41557676	3	SM_37
A			
A	32953580	3	OM_42
B	17911502	3	AD_42
B			
B	15478104	3	AD_37
B			
B	10705805	3	SM_42
B			
B	10670995	3	OM_37

Table S71: Uniprot accession number=F7CL80

Means with the same letter are not significantly different.			
Duncan Grouping	Mean	N	GRP
A	3456115	3	OM_42
B	2119389	3	AD_42
B			
B	2029792	3	SM_37
B			
B	2004331	3	AD_37
B			
B	1992874	3	OM_37
B			
B	1706747	3	SM_42

Table S72: Uniprot accession number=F7BXA6

Means with the same letter are not significantly different.				
Duncan Grouping		Mean	N	GRP
	A	15316297	3	OM_42
	A			
B	A	12317519	3	AD_37
B				
B		10815653	3	SM_37
B				

Means with the same letter are not significantly different.				
Duncan Grouping		Mean	N	GRP
B		10265261	3	OM_37
B				
B		10209812	3	AD_42
B				
B		9064235	3	SM_42

Table S73: Uniprot accession number=F7CAN3

Means with the same letter are not significantly different.			
Duncan Grouping	Mean	N	GRP
A	179168	3	SM_42
A			
A	178677	3	OM_37
A			
A	171137	3	AD_42
A			
A	150772	3	AD_37
A			
A	133845	3	OM_42
A			
A	122470	3	SM_37

Table S74: Uniprot accession number=F7C4W2

Means with the same letter are not significantly different.				
Duncan Grouping		Mean	N	GRP
	A	893317	3	OM_42
	A			
	A	810298	3	AD_37
	A			
	A	787589	3	OM_37
	A			
	A	785907	3	SM_42
	A			
B	A	718576	3	SM_37
B				
B		572408	3	AD_42

Table S75: Uniprot accession number=F7APL8

Means with the same letter are not significantly different.			
Duncan Grouping	Mean	N	GRP
A	4086773	3	AD_37
A			
A	4062185	3	OM_42

Means with the same letter are not significantly different.			
Duncan Grouping	Mean	N	GRP
A			
A	3809597	3	SM_42
A			
A	3557929	3	OM_37
A			
A	3300668	3	SM_37
A			
A	3053522	3	AD_42

Table S76: Uniprot accession number=A0A3Q2HEJ2

Means with the same letter are not significantly different.			
Duncan Grouping	Mean	N	GRP
A	3385171	3	AD_42
A			
A	3332330	3	OM_37
A			
A	3246521	3	SM_37
A			
A	3234086	3	OM_42
A			
A	2848667	3	AD_37
B	1911953	3	SM_42

Table S77: Uniprot accession number=A0A3Q2I4J6

Means with the same letter are not significantly different.				
Duncan Grouping		Mean	N	GRP
	A	2131470	3	AD_37
	B	1576487	3	OM_42
	B			
C	B	1274580	3	SM_42
C	B			
C	B	1269314	3	OM_37
C	B			
C	B	1119846	3	SM_37
C				
C		963162	3	AD_42

Table S78: Uniprot accession number=F6TMS0

Means with the same letter are not significantly different.					
Duncan Grouping			Mean	N	GRP
	A		3092276	3	SM_42
	A				
B	A		2793020	3	AD_37
B	A				
B	A		2783303	3	SM_37
B	A				
B	A	C	2632013	3	OM_42
B		C			
B		C	2234795	3	OM_37
		C			
		C	1999579	3	AD_42

Table S79: Uniprot accession number=F6XL78

Means with the same letter are not significantly different.				
Duncan Grouping		Mean	N	GRP
	A	1165902	3	OM_37
	B	654960	3	AD_37
	B			
C	B	486536	3	SM_37
C				
C		404024	3	SM_42
C				
C		329312	3	OM_42
	D	97529	2	AD_42

Table S80: Uniprot accession number=F6UJZ8

Means with the same letter are not significantly different.				
Duncan Grouping		Mean	N	GRP
	A	235034	3	OM_37
	A			
B	A	159969	3	OM_37
B	A			
B	A	146958	3	SM_37
B	A			
B	A	135838	3	AD_37
B				
B		86867	2	AD_42
B				
B		67962	3	SM_42

Table S81: Uniprot accession number=F7DBF8

Means with the same letter are not significantly different.				
Duncan Grouping		Mean	N	GRP
	A	171701	3	OM_37
	A			
	A	149002	3	SM_37
	A			
	A	148595	3	AD_37
	A			
	A	133870	3	OM_42
	A			
B	A	96229	3	SM_42
B				
B		0	2	AD_42

Table S82: Uniprot accession number=A0A5F5PF14

Means with the same letter are not significantly different.			
Duncan Grouping	Mean	N	GRP
A	191256	3	SM_37
A			
A	178664	3	AD_37
A			
A	159736	3	OM_37
A			
A	126713	2	AD_42
A			
A	124157	3	OM_42
A			
A	42542	3	SM_42

Table S83: Uniprot accession number=H9GZW3

Means with the same letter are not significantly different.				
Duncan Grouping		Mean	N	GRP
	A	255524	3	OM_42
	A			
	A	247386	3	OM_37
	B	147011	3	SM_42
	B			
	B	139228	2	AD_42
	B			
C	B	102262	3	SM_37

Means with the same letter are not significantly different.				
Duncan Grouping		Mean	N	GRP
C				
C		51250	3	AD_37

Table S84: Uniprot accession number=A0A3Q2LA78

Means with the same letter are not significantly different.					
Duncan Grouping			Mean	N	GRP
	A		61102	3	OM_42
	A				
B	A		52136	3	AD_37
B	A				
B	A	C	32659	3	OM_37
B		C			
B		C	23001	3	SM_37
B		C			
B		C	20986	3	SM_42
		C			
		C	20198	2	AD_42

Table S85: Uniprot accession number=A0A3Q24KJ26

Means with the same letter are not significantly different.				
Duncan Grouping		Mean	N	GRP
	A	2131470	3	OM_42
	B	1576487	3	AD_37
	B			
C	B	1274580	3	OM_37
C	B			
C	B	1269314	3	SM_42
C	B			
C	B	1119846	3	SM_37
C				
C		963162	2	AD_42

Table S86: Uniprot accession number=F7DBU9

Means with the same letter are not significantly different.					
Duncan Grouping			Mean	N	GRP
	A		209621	3	OM_37
	A				
B	A		165996	3	OM_42
B	A				
B	A	C	137024	3	SM_37
B		C			

Means with the same letter are not significantly different.					
Duncan Grouping			Mean	N	GRP
B		C	108479	3	AD_37
		C			
	D	C	70095	3	SM_42
	D				
	D		18368	2	AD_42

Table S87: Uniprot accession number=A0A3Q2LT78

Means with the same letter are not significantly different.				
Duncan Grouping		Mean	N	GRP
	A	330855	3	SM_37
	B	187212	3	AD_37
	B			
C	B	113324	3	SM_42
C	B			
C	B	112371	3	OM_37
C				
C		67642	3	OM_42
C				
C		26465	2	AD_42

Table S88: Uniprot accession number=A0A3Q2H8V2

Means with the same letter are not significantly different.			
Duncan Grouping	Mean	N	GRP
A	41192	3	SM_42
A			
A	36195	2	AD_42
A			
A	31823	3	AD_37
A			
A	30483	3	OM_42
A			
A	25732	3	OM_37
A			
A	15587	3	SM_37

Table S89: Uniprot accession number=F7CE39

Means with the same letter are not significantly different.				
Duncan Grouping		Mean	N	GRP
	A	93954	3	OM_37
	B	56104	3	SM_42

Means with the same letter are not significantly different.				
Duncan Grouping		Mean	N	GRP
	B			
	B	55997	3	OM_42
	B			
C	B	47567	2	AD_42
C	B			
C	B	38453	3	AD_37
C				
C		22825	3	SM_37
